# Ethnic and income-related inequalities in mental health among adolescents: a cross-sectional study in Bradford, UK

**DOI:** 10.1136/bmjph-2025-004528

**Published:** 2026-09-15

**Authors:** Dan Lewer, Sunil Bhopal, Helen Dale, Mohammed Hammad, Rosemary R C McEachan, Matthias Pierce, John Wright

**Affiliations:** 1 Born in Bradford, Bradford Institute for Health Research, Bradford, UK; 2 Department of Epidemiology and Public Health, University College London, London, UK; 3 Population Health Improvement (PHI-UK), Cambridge, UK; 4 Faculty of Epidemiology & Population Health, London School of Hygiene & Tropical Medicine, London, UK; 5 School of Population Health Sciences, University of Edinburgh, Edinburgh, UK; 6 Centre for Women’s Mental Health, Faculty of Medicine Biology and Health, University of Manchester, Manchester, UK

**Keywords:** Mental Health, Community Health, Public Health, Sociodemographic Factors

## Abstract

**Introduction:**

Poverty is a well-established determinant of adolescent mental health, but associations between ethnicity and mental health are less clear. We quantified inequalities in mental health and well-being among adolescents in Bradford, UK.

**Methods:**

We conducted a cross-sectional analysis of the Born in Bradford birth cohort, using surveys from 2023/2024 (ages 12–15). Participants completed validated measures of anxiety and depression (Revised Child Anxiety and Depression Scale 25), well-being (Short Warwick-Edinburgh Mental Well-being Scale), emotional and behavioural problems (Strengths and Difficulties Questionnaire), loneliness (UCLA Loneliness Scale 4), eating-related problems (Eating Disorders Examination Questionnaire-Short) and psychosis-like experiences (Psychosis-Like Symptoms Scale 8). We used linear regression to estimate associations with ethnicity and family income (measured by free school meals eligibility).

**Results:**

We included 9496 participants (median age 13.9 years; 52.1% female; 40.9% Pakistani ethnicity; 34.4% white British ethnicity; 24.6% other ethnicities; and 62.0% living in the most deprived quintile of neighbourhoods in England). Compared with adolescents of white British ethnicity, those of Pakistani ethnicity had fewer anxiety and depression symptoms (Cohen’s d=−0.31, 95% CI −0.37 to −0.24); higher well-being (d=0.15, 95% CI 0.10 to 0.20), fewer emotional and behavioural problems (d=−0.40; 95% CI −0.48 to −0.32); less loneliness (d=−0.22, 95% CI −0.26 to −0.17), fewer eating-related problems (d=−0.07; 95% CI −0.11 to −0.02) and no evidence of a difference in psychosis-like experiences (d=−0.07; 95% CI −0.17 to 0.02). Estimates for other minority ethnic groups were less precise but generally suggested better mental health than white British adolescents. Adolescents eligible for free school meals had poorer outcomes across all measures: anxiety and depression (d=0.13; 95% CI 0.07 to 0.18); well-being (d=−0.17; 95% CI −0.22 to −0.12); loneliness (d=0.09; 95% CI 0.04 to 0.14); emotional and behavioural problems (d=0.14; 95% CI 0.06 to 0.22); eating-related problems (d=0.06; 95% CI 0.01 to 0.11); and psychosis-like experiences (d=0.10; 95% CI 0.01 to 0.20). In exploratory sex-stratified analyses, girls reported poorer mental health and ethnic differences were wider among girls than boys.

**Conclusions:**

Poverty was consistently associated with poorer adolescent mental health. Despite higher deprivation, Pakistani adolescents reported substantially better outcomes than white British adolescents, particularly for emotional and behavioural problems.

**WHAT IS ALREADY KNOWN ON THIS TOPIC**

**WHAT THIS STUDY ADDS**

- In a city in the North of England with a large population of Pakistani heritage, adolescents of Pakistani ethnicity reported substantially better mental health and well-being than white British adolescents. This difference was observed across a wide range of outcomes including anxiety, depression, well-being, behavioural and emotional problems, loneliness and eating-related problems, and was despite greater average material deprivation among adolescents of Pakistani heritage.

**HOW THIS STUDY MIGHT AFFECT RESEARCH, PRACTICE OR POLICY**

- Established minority ethnic communities may possess protective factors related to family, neighbourhood and religion; leading to lower risk of mental health problems in adolescents. This finding may inform investment in resilient social institutions that are relevant to adolescents, such as youth groups; sporting, artistic and cultural opportunities; and religious groups.

## Introduction

 Adolescent mental health has become a major public health priority in many high-income countries, with reported increases in anxiety, depression and other forms of psychological distress over the past two decades.^1,2^ In the UK, some evidence suggests that these increases have not occurred uniformly across ethnic groups, with greater increases in white British adolescents.^3^

Proposed drivers of a general increase in adolescent mental health problems include changes to public services; academic pressure; societal expectations and parenting styles; sleep quality; social media and smartphone use; and the effects of COVID-19 lockdowns on social and education opportunities.^1,3,4^ Adolescents from minority ethnic backgrounds may be less exposed to some of these contemporary risk factors, or may benefit from protective factors such as stronger family support, community cohesion and religious or cultural connectedness. Alternatively, in the context of changing societal understandings of mental health,^5^ observed differences between ethnic groups may partly reflect variations in how symptoms are perceived and reported.

National datasets have important limitations for studying associations between ethnicity and adolescent mental health. Ethnic groups are unevenly distributed geographically, so observed inequalities may partly reflect place-based factors such as school environments, neighbourhood deprivation or local service provision. Many national studies also lack statistical power due to small numbers of participants from minority groups, and the use of broad ethnic categories can obscure important heterogeneity. For example, in the UK the label ‘Asian’ often combines individuals of Pakistani, Indian and Bangladeshi heritage, despite evidence that mental health outcomes differ across these groups.^6^

Bradford is an ethnically diverse city in northern England with a large population of Pakistani heritage. The Born in Bradford study, a birth cohort in which index participants are currently in adolescence, provides a unique opportunity to examine inequalities in this setting, capturing a wide range of validated measures of mental health and well-being. In this study, we aimed to describe inequalities in self-reported adolescent mental health by ethnicity and socio-economic status.

## Methods and materials

### Design

We conducted a cross-sectional study estimating the association between sociodemographic factors and mental health and well-being among adolescents. This report follows the Strengthening the Reporting of Observational Studies in Epidemiology (STROBE) guidelines (see online supplemental file 1 section 7 for STROBE checklist).^7^

### Data source and participants

**Figure 1:**
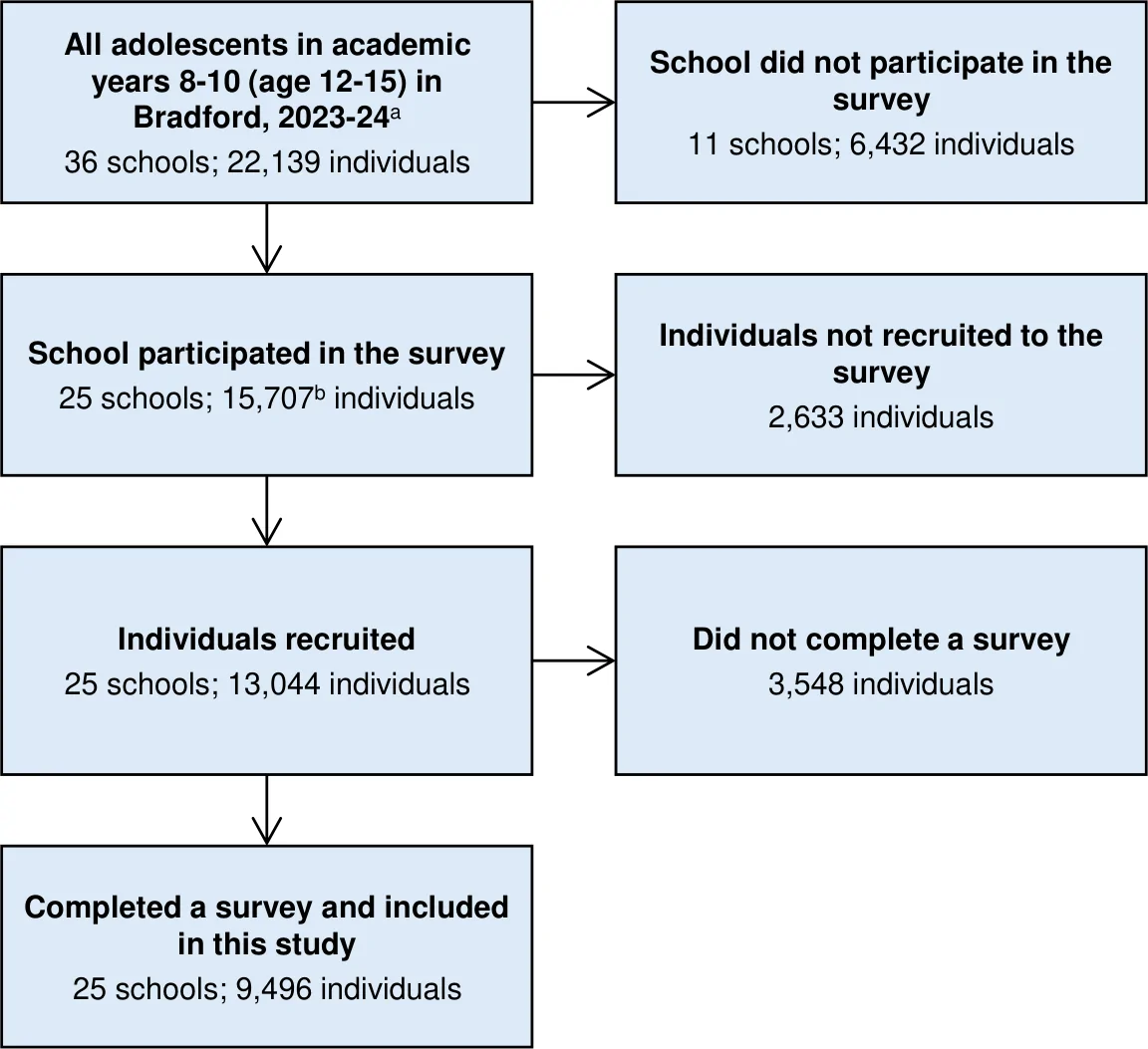
Recruitment flow chart. (a) The headcount of students in years 8–10 in schools in Bradford is taken from the Department for Education. (b) We accessed the headcount of students in years 8–10 in 2023/2024 from the local authority (https://datahub.bradford.gov.uk/datasets/education/bradford-pupils/). This data covered 24/25 schools, totalling 15 079 pupils. We assumed that the final school had the mean number of pupils.

We used data from Born in Bradford’s ‘Age of Wonder’ study. Born in Bradford is a birth cohort study in Bradford, UK, in which participants were recruited at birth between 2007 and 2011.^8,9^ ‘Age of Wonder’ is the follow-up at age 12–15, with surveys conducted in classrooms in secondary schools, supervised by a research assistant. Surveys included a wide range of topics including mental health and well-being. We used data from surveys conducted in academic year 2023/2024, between 1 September 2023 and 31 July 2024. The number of participants at each stage of recruitment is summarised in figure 1. Considering the local population of students in academic years 8–10 as the target population, we included 9496/22 139 (42.9%) in this study. Out of 12 643 students not included, the largest source of non-response was school-level (6432/12 643; 50.9%); with the remainder either not consenting to participate or not completing a survey on the day, typically due to absence from school.

### Outcomes

We studied six self-reported measures of mental health and well-being: (1) The Revised Child Anxiety and Depression Scale 25 (RCADS25),^10^ which is designed to measure symptoms of depression and anxiety; (2) The Short Warwick-Edinburgh Mental Well-being Scale (SWEMWBS),^11^ which is designed to measure well-being; (3) The UCLA Loneliness Scale 4 (ULS4)^12^; (4) The Strengths and Difficulties Questionnaire (SDQ),^13^ which is designed to measure emotional and behavioural problems, with subscales measuring problems related to emotions, peers, conduct and hyperactivity; (5) The Eating Disorders Examination Questionnaire-Short (EDE-QS),^14^ which is designed to measure eating-related problems; (6) the Psychosis-Like Symptoms (PLIKS) questionnaire.^15^ For each measure, we calculated a summary score following standard procedures. Some measures were administered within specific year groups. The outcomes measures and full questionnaires are provided in online supplemental files 2, 3 under ‘AgeOfWonder_Questionnaire1.pdf’ and ‘AgeOfWonder_Questionnaire2.pdf’.

### Exposures

The two exposures were ethnicity and eligibility for free school meals, both provided by participating schools.

Schools collect pupil ethnicity from parents or carers. We defined ethnicity using the UK Office for National Statistics classification 8a^16^ following the standard recording practices by schools in the UK. We grouped ‘white: Irish’ and ‘white: Gypsy or Irish Traveller, Roma or other white’ into ‘other white’ due to small numbers of students in these categories; and split ‘Asian, Asian British or Asian Welsh’ into ‘Pakistani’ and ‘other Asian’ due to the large number of students of Pakistani heritage. Consistent with other studies in this field,^17^ we used ‘white British’ as the reference category.

We used eligibility for free school meals as a proxy for family income. In England, families pay for school meals, with eligibility for free school meals determined by receipt of means-tested benefits.^18^ Families must apply to their local authority for free school meals. Government research in 2013 estimated that 11% of pupils nationally who meet these criteria have not claimed eligibility; and 9% in Bradford.^19^

### Other variables

To adjust for potential confounding, we included (1) age in months at the date of the survey, calculated from the participant’s date of birth and the date of survey completion; (2) the participant’s sex (male/female) as reported by the school; and (3) the season when the survey was completed (autumn, winter, spring or summer). We chose a limited set of potentially confounding variables to avoid adjusting for mediators, since most observable differences between ethnicity or income groups are best interpreted as the consequences rather than causes of these exposures.

For descriptive purposes, we also reported (1) the Index of Multiple Deprivation 2019^20^ for the participant’s home address. This is an area-based composite measure of deprivation based on Lower Super Output Areas (neighbourhoods of approximately 1500 residents); (2) the population density of the Lower Super Output Area of the participant’s home address; (3) Special Educational Needs provision, as reported by schools. Special Educational Needs provision was grouped as no recorded needs, a known additional need or an ‘Education, Health and Care Plan’, which follows a formal assessment and places an obligation on the local authority to provide additional support.

### Statistical analysis

We described the outcomes using histograms. We then used linear regression to estimate the mean difference in outcome scores between exposure groups. In each model, the dependent variable was a measure of mental health and well-being; the main independent variable was either ethnicity or eligibility for free school meals; and confounding variables were also included. For ethnicity, we used a reference category of ‘White British’ for consistency with other studies in the UK.^17^ In some of these analyses we observed moderate heteroskedasticity and therefore used a heteroskedasticity-robust sandwich estimator to estimate the standard error, using the HC1 formula.

We divided the mean differences estimated from these models by the pooled standard deviation of the outcome to estimate standardised mean differences, sometimes known as ‘Cohen’s d’. For all outcomes except SWEMWBS, higher scores indicated worse mental health and well-being; therefore, a positive effect indicated better mental health and well-being in the reference group.

Missing data were principally in the outcome measures, ranging from 10.8% of participants with no EDE-QS items to 23.6% with no RCADS25 items (see online supplemental file 1 section 1 for a breakdown of missing data). Missing data was primarily due to the participant being absent from school when the relevant section of the survey was administered. Exposure measures and covariates had minimal missing data. We used a bootstrap-based multiple imputation algorithm to generate 20 complete datasets. Individual survey items were included in this procedure, and we calculated summary scales within imputed datasets. This allowed partially completed outcome instruments to contribute to imputation of remaining items. We repeated the procedures above for each complete dataset and combined the results using Rubin’s rules.^21^ We compared our main results to a complete case analysis (see online supplemental file 1 section 5) and observed only minor differences.

In exploratory analysis, we included an interaction between sex and the exposures (ethnicity or free school meal eligibility), then predicted sex-stratified results. We limited the interaction between sex and ethnicity to participants of white British and Pakistani ethnicities due to limited power to estimate interactions for other ethnic groups.

All analysis was done using R 4.4.1^22^ with packages ‘sandwich’ for the robust sandwich estimator^23^ and ‘Amelia2’ for multiple imputation.^24^ Analysis code is available at https://github.com/danlewer/aow_adolescent_mh.

### Role of adolescents in this study

Coproduction and engagement with young people, families and communities are central to Born in Bradford. This has informed research priorities for the adolescent follow-up, including a focus on mental health. Adolescents have also helped to design our methods and data collection procedures, and to help disseminate our findings.^25^

### Role of the funding source

The funder of the study had no role in study design, data collection, data analysis, data interpretation or writing of the report.

## Results

### Characteristics of participants

The study included 9496 participants with a median age of 13.9 years (IQR 13.2–14.6). 4952 were female (52.1%); 3885 (40.9%) were of Pakistani ethnicity; 3271 (34.4%) were white British; 2340 (24.6%) were of other ethnicities; and 5888 (62.0%) lived in the most deprived quintile of neighbourhoods nationally. The sample was approximately representative of adolescents in Bradford in terms of sex, urbanicity, special educational needs, free school meal eligibility, ethnicity and neighbourhood deprivation. Characteristics of the sample are summarised in table 1. The distribution of outcome measures are shown graphically in online supplemental file 1 section 3 and online supplemental file 1 section 4.

**Table 1:** Sample characteristics, compared to adolescents in Bradford

Variable	Level	Study sample N (%) Median [IQR]	Adolescents in Bradford % Median [IQR**]**
Total		9496 (100.0)	100.0
Age at survey completion (years)		13.9 [13.2–14.6]	–
Season of survey completion	Autumn 2023	1908 (20.1)	–
	Winter 2023/24	3319 (35.0)	–
	Spring 2024	1938 (20.4)	–
	Summer 2024	2331 (24.5)	–
Sex*	Female	4952 (52.1)	48.9
	Male	4544 (47.9)	51.1
National curriculum year group*	8	3385 (35.6)	33.4
	9	3366 (35.4)	33.6
	10	2745 (28.9)	33.0
Population density (residents/km^2^)†		4984 [3291–7202]	4806 [2868–7259]
SEN*	None	7700 (81.1)	80.2
	SEN	1225 (12.9)	14.9
	EHCP	137 (1.4)	4.9
	Missing	434 (4.6)	–
Eligible for free school meals*	No	6737 (70.9)	66.5
	Yes	2759 (29.1)	33.5
Ethnicity*	Asian and Asian British	4589 (48.3)	44.2
	*Of which Pakistani*	*3885* (*40.9*)	37.3
	*Of which other Asian*	*704* (*7.4*)	6.9
	White	3705 (39.0)	43.1
	*Of which white British*	*3271* (*34.4*)	37.7
	*Of which other white*	*434* (*4.6*)	5.5
	Black and black British	341 (3.6)	2.9
	Mixed ethnicities	498 (5.2)	5.8
	Other	171 (1.8)	1.8
	Missing	192 (2.0)	2.1
Index of Multiple Deprivation 2019 quintile†	1 (most deprived)	5888 (62.0)	57.1
	2	1909 (20.1)	19.5
	3	656 (6.9)	9.4
	4	433 (4.6)	7.8
	5 (least deprived)	603 (6.4)	6.2
	Missing	7 (0.1)	–

* Data for adolescents in Bradford are from the Department for Education 2023/2024 school census for pupils in year 8–10 at state-funded secondary schools in England, accessed via https://explore-education-statistics.service.gov.uk/.

† Population density and Index of Multiple Deprivation of home address are calculated from ONS data for 12–15 years old in Bradford, using data on population by single-year-of-age, land area and Index of Multiple Deprivation, at Lower Level Super Output Area level.

EHCP, Education, Health and Care Plan; ONS, Office for National Statistics; SEN, Special Educational Needs.

Compared with adolescents of the same age across England, the sample included a greater proportion from minority ethnic backgrounds (especially of Pakistani ethnicity); was more urban (ie, lived in areas with higher population density); and was more likely to live in deprived areas (see online supplemental file 1 section 2).

Socio-economic status, as measured by eligibility for free school meals and the Index of Multiple Deprivation, varied by ethnicity. Participants of ‘other’, ‘mixed’ and ‘other white’ ethnicities were most likely to be eligible for free school meals. White British participants were substantially less likely to live in the most deprived quintile of neighbourhoods than participants of other ethnic groups (table 2).

**Table 2:** Socio-economic status of participants by ethnic group

Ethnicity	Total participants	Eligible for free school meals (p<0.001)*		Living in the most deprived quintile of neighbourhoods nationally† (p<0.001)*	
		Number	Per cent (95% CI)	Number	Per cent (95% CI)
Total	9496	2759	29.1 (28.1 to 30.0)	5888	62.0 (61.0 to 63.0)
Pakistani	3885	1069	27.5 (26.1 to 29.0)	2932	75.5 (74.1 to 76.8)
White British	3271	843	25.8 (24.3 to 27.3)	1282	39.2 (37.5 to 40.9)
Other Asian	704	176	25.0 (21.9 to 28.4)	482	68.5 (64.9 to 71.9)
Mixed	498	228	45.8 (41.4 to 50.3)	309	62.0 (57.6 to 66.3)
Other white	434	163	37.6 (33.0 to 42.3)	346	79.7 (75.6 to 83.3)
Black	341	113	33.1 (28.2 to 38.4)	274	80.4 (75.6 to 84.4)
Unknown	192	59	30.7 (24.4 to 37.9)	127	66.1 (58.9 to 72.7)
Other	171	108	63.2 (55.4 to 70.3)	136	79.5 (72.6 to 85.2)

* Based on χ² tests.

† Based on the Index of Multiple Deprivation 2019.

**Figure 2:**
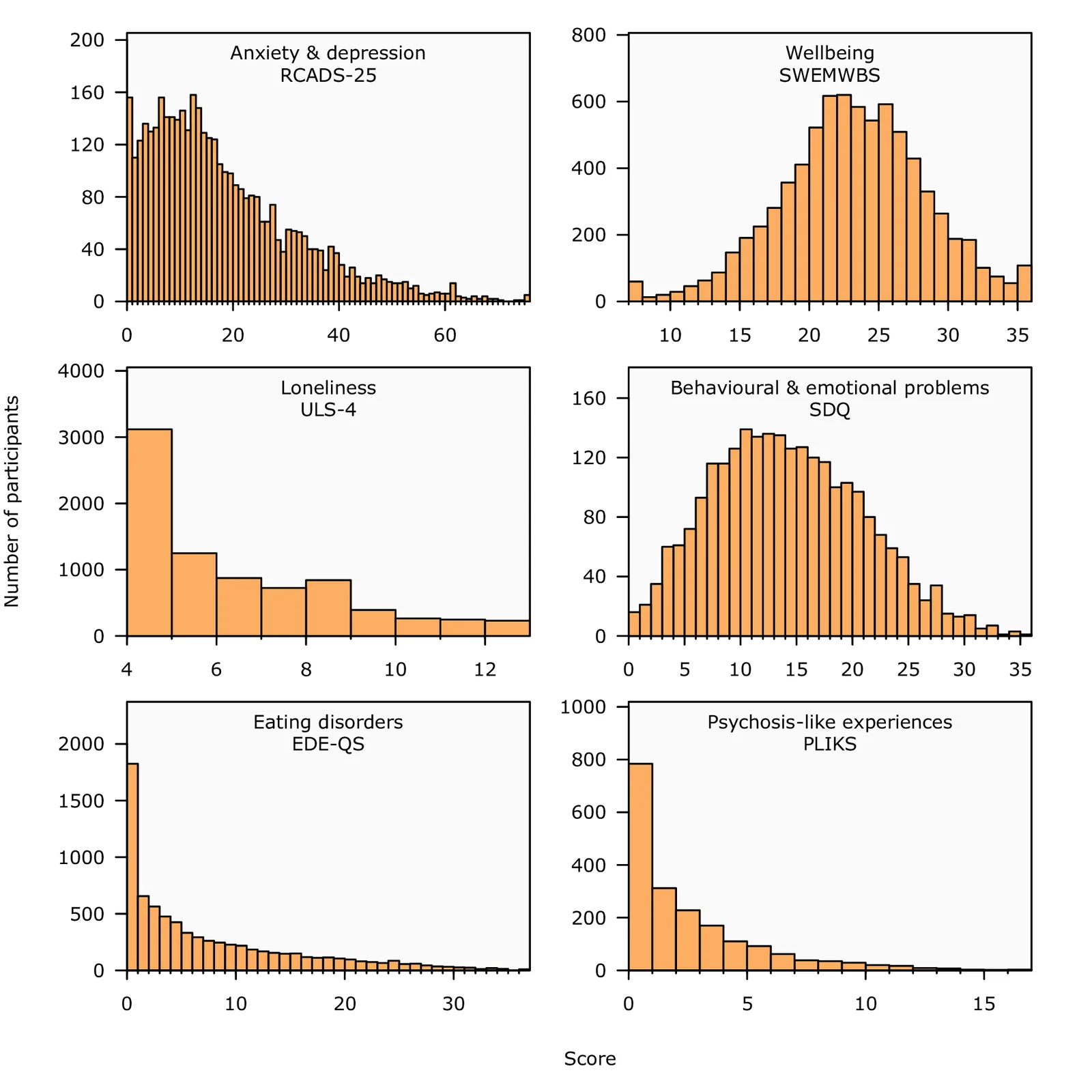
Histograms of mental health and well-being measures. EDE-QS, Eating Disorders Examination Questionnaire-Short; PLIKS, Psychosis-Like Symptoms; RCADS-25, Revised Child Anxiety and Depression Scale 25; SDQ, Strengths and Difficulties Questionnaire; SWEMWBS, Short Warwick-Edinburgh Mental Well-being Scale; ULS-4, UCLA Loneliness Scale 4.

Mental health measures had a variety of distributions. RCADS25, ULS4, EDE-QS and PLIKS were right-skewed, while SWEMWBS and SDQ had a more symmetrical distribution (figure 2).

### Associations between ethnicity, socio-economic status and mental health

**Figure 3:**
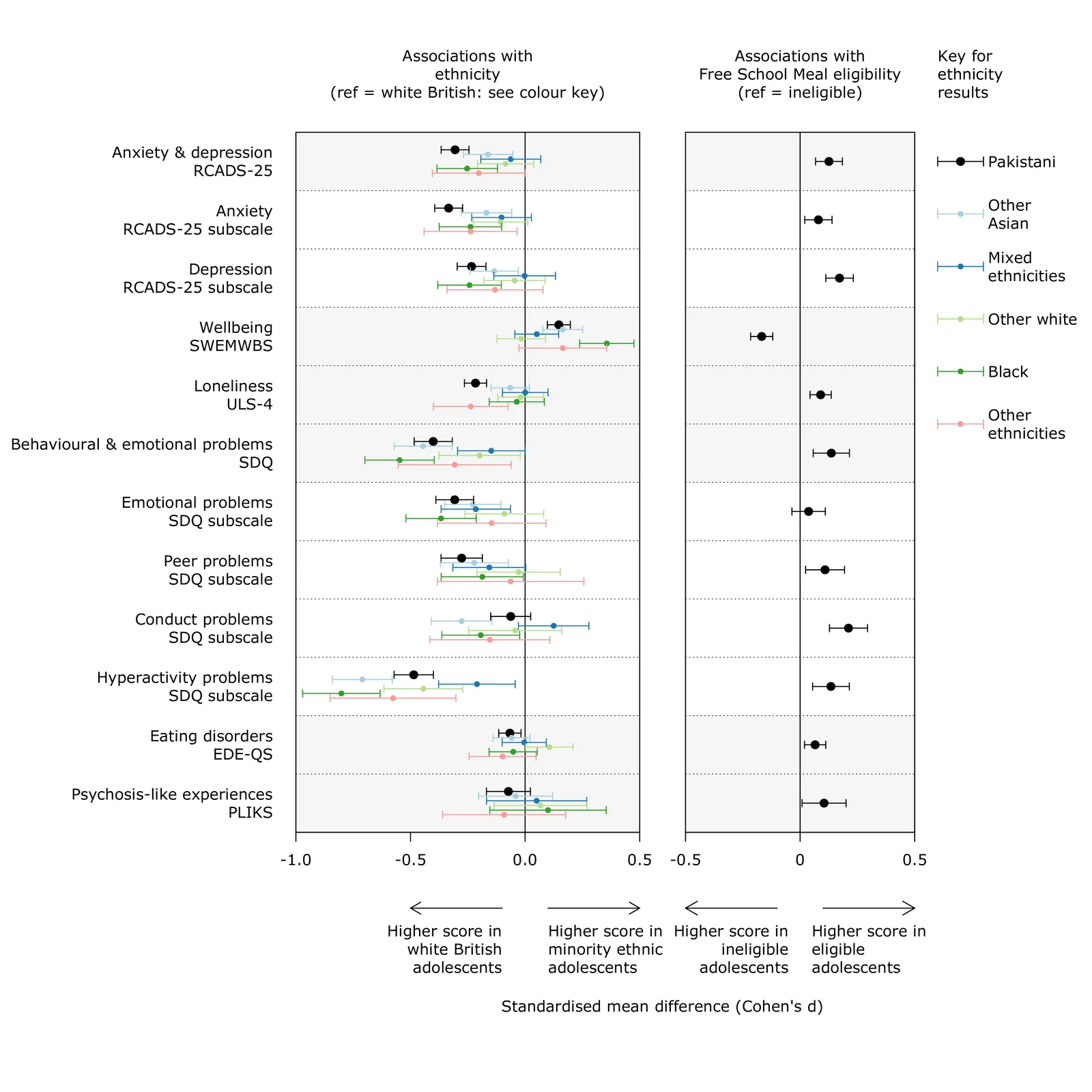
Adolescent mental health and well-being by ethnicity and eligibility for free school meals: standardised mean differences and 95% CIs. For all measures except SWEMWBS, a higher score indicates greater symptoms or worse mental health. For SWEMWBS, a higher score indicates better mental well-being. EDE-QS, Eating Disorders Examination Questionnaire-Short; RCADS-25, Revised Child Anxiety and Depression Scale 25; SDQ, Strengths and Difficulties Questionnaire; SWEMWBS, Short Warwick-Edinburgh Mental Well-being Scale.

We found strong evidence that ethnicity was associated with mental health and well-being. Compared with adolescents of white British ethnicity, those of Pakistani ethnicity had fewer anxiety and depression symptoms measured using RCADS25 (Cohen’s d=−0.31, 95% CI −0.37 to −0.24); higher well-being measured using SWEMWBS (d=0.15, 95% CI 0.10 to 0.20), fewer emotional and behavioural problems measured using SDQ (d=−0.40; 95% CI −0.48 to −0.32); less loneliness measured using ULS4 (d=−0.22, 95% CI −0.26 to −0.17), fewer eating-related problems measured using EDE-QS (d=−0.07; 95% CI −0.11 to −0.02) and no evidence of a difference in psychosis-like experiences measured using PLIKS (d=−0.07; 95% CI −0.17 to 0.02). When comparing adolescents of white British and Pakistani ethnicities on subscales, we found an especially large difference on the hyperactivity subscale of SDQ (d=−0.49; 95% CI −0.57 to −0.40). Estimates for other minority ethnic groups were less precise but generally suggested better mental health than white British adolescents. We did not find evidence that any minority ethnic groups had higher scores for mental health measures or lower well-being, though in many cases CIs crossed zero (figure 3 and table 3).

**Table 3:** Adolescent mental health and well-being in groups defined by ethnicity and eligibility for free school meals: standardised mean difference (95% CIs)

Instrument	Associations with ethnicity (ref=white British)						Eligible for free school meals (ref=not eligible)
	Pakistani	Other Asian	Mixed	White other	Black	Other	
RCADS25	−0.31 (−0.37 to −0.24)	−0.16 (−0.27 to −0.05)	−0.06 (−0.19 to 0.07)	−0.08 (−0.21 to 0.04)	−0.25 (−0.38 to −0.12)	−0.20 (−0.40 to 0.00)	0.13 (0.07 to 0.18)
RCADS25 anxiety subscale	−0.33 (−0.39 to −0.27)	−0.17 (−0.28 to −0.06)	−0.10 (−0.23 to 0.03)	−0.11 (−0.23 to 0.01)	−0.24 (−0.37 to −0.10)	−0.24 (−0.44 to −0.03)	0.08 (0.02 to 0.14)
RCADS25 depression subscale	−0.23 (−0.30 to −0.17)	−0.13 (−0.24 to −0.03)	0.00 (−0.14 to 0.13)	−0.05 (−0.18 to 0.09)	−0.24 (−0.38 to −0.10)	−0.13 (−0.34 to 0.08)	0.17 (0.11 to 0.23)
SWEMWBS	0.15 (0.10 to 0.20)	0.16 (0.08 to 0.25)	0.05 (−0.04 to 0.15)	−0.02 (−0.12 to 0.09)	0.36 (0.24 to 0.48)	0.16 (−0.03 to 0.36)	−0.17 (−0.22 to −0.12)
ULS4	−0.22 (−0.26 to −0.17)	−0.07 (−0.15 to 0.02)	0.00 (−0.10 to 0.10)	−0.02 (−0.12 to 0.08)	−0.04 (−0.16 to 0.08)	−0.24 (−0.40 to −0.07)	0.09 (0.04 to 0.14)
SDQ	−0.40 (−0.48 to −0.32)	−0.45 (−0.57 to −0.32)	−0.15 (−0.29 to 0.00)	−0.20 (−0.38 to −0.02)	−0.55 (−0.70 to −0.40)	−0.31 (−0.55 to −0.06)	0.14 (0.06 to 0.22)
SDQ emotional subscale	−0.31 (−0.39 to −0.22)	−0.23 (−0.35 to −0.11)	−0.22 (−0.37 to −0.06)	−0.09 (−0.26 to 0.08)	−0.37 (−0.52 to −0.21)	−0.15 (−0.38 to 0.09)	0.04 (−0.04 to 0.11)
SDQ peer subscale	−0.28 (−0.37 to −0.19)	−0.22 (−0.37 to −0.07)	−0.16 (−0.32 to 0.00)	−0.03 (−0.21 to 0.15)	−0.19 (−0.37 to −0.01)	−0.06 (−0.38 to 0.26)	0.11 (0.02 to 0.19)
SDQ conduct subscale	−0.06 (−0.15 to 0.02)	−0.28 (−0.41 to −0.14)	0.12 (−0.03 to 0.28)	−0.04 (−0.25 to 0.16)	−0.19 (−0.36 to −0.02)	−0.15 (−0.42 to 0.11)	0.21 (0.13 to 0.29)
SDQ hyperactivity subscale	−0.49 (−0.57 to −0.40)	−0.71 (−0.84 to −0.58)	−0.21 (−0.38 to −0.04)	−0.44 (−0.62 to −0.27)	−0.80 (−0.97 to −0.63)	−0.58 (−0.85 to −0.30)	0.13 (0.05 to 0.21)
EDEQS	−0.07 (−0.11 to −0.02)	−0.06 (−0.14 to 0.02)	0.00 (−0.10 to 0.09)	0.11 (0.00 to 0.21)	−0.05 (−0.16 to 0.05)	−0.10 (−0.24 to 0.05)	0.07 (0.02 to 0.11)
PLIKS	−0.07 (−0.17 to 0.02)	−0.04 (−0.20 to 0.12)	0.05 (−0.17 to 0.27)	0.07 (−0.14 to 0.27)	0.10 (−0.15 to 0.35)	−0.09 (−0.36 to 0.18)	0.10 (0.01 to 0.20)

For all measures except SWEMWBS, a higher score indicates greater symptoms or worse mental health. For SWEMWBS, a higher score indicates better mental well-being. The colours in the table represent the size of association, in which red represents worse mental health and well-being in the comparison group (or better mental health and well-being in the reference group), and green represents better mental health and well-being in the comparison group. For example, green cells in the ethnicity columns would suggest better mental health and well-being among the relevant group of minority ethnic adolescents; while green cells in the free school meal eligiblity column would represent better mental health among adolescents who are eligible for free school meals.

EDEQS, Eating Disorders Examination Questionnaire-Short; PLIKS, Psychosis-Like Symptoms; RCADS25, Revised Child Anxiety and Depression Scale 25; SDQ, Strengths and Difficulties Questionnaire; SWEMWBS, Short Warwick-Edinburgh Mental Well-being Scale; ULS4, UCLA Loneliness Scale 4.

Adolescents eligible for free school meals had poorer outcomes than those who were not eligible across all measures: anxiety and depression (d=0.13; 95% CI 0.07 to 0.18); well-being (d=−0.17; 95% CI −0.22 to −0.12); loneliness (d=0.09, 95% CI 0.04 to 0.14), emotional and behavioural problems (d=0.14; 95% CI 0.06 to 0.22), eating-related problems (d=0.06; 95% CI 0.01 to 0.11); and psychosis-like experiences (d=0.10; 95% CI 0.01 to 0.20). When comparing adolescents of white British and Pakistani ethnicities on subscales, we found an especially large difference on the conduct-related subscale of SDQ (d=0.21, 95% CI 0.13 to 0.29).

**Figure 4:**
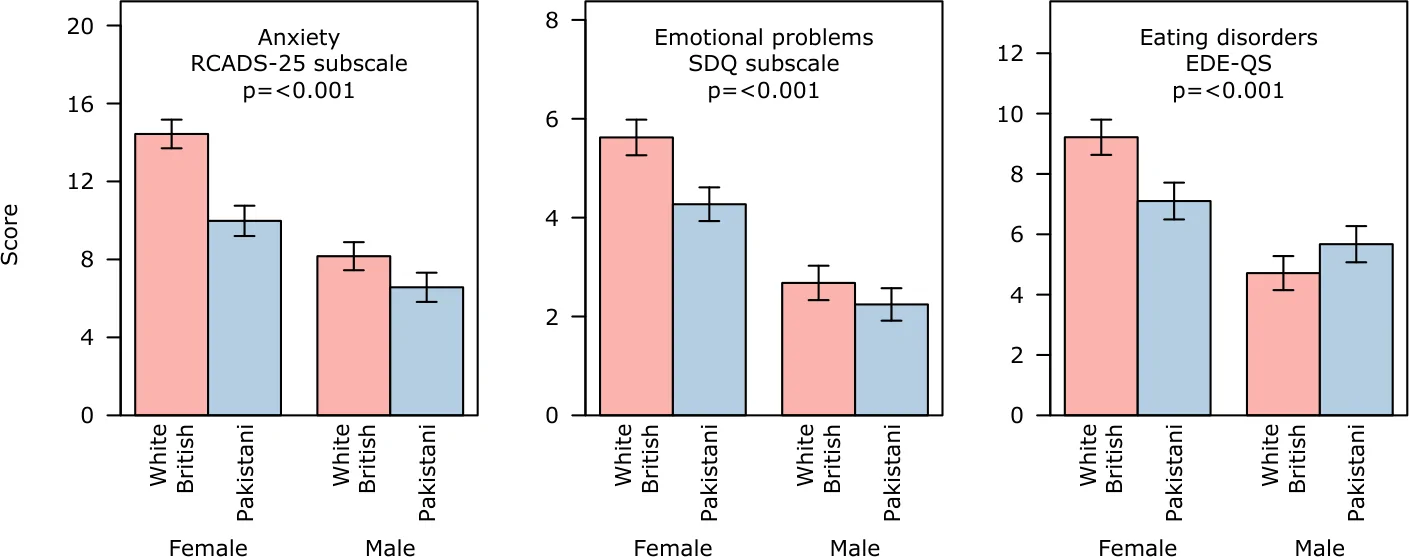
Sex-specific mental health scores predicted from regression models with an interaction between sex and ethnicity, for selected measures of mental health. P values relate to the interaction term between ethnicity and sex. EDE-QS, Eating Disorders Examination Questionnaire-Short; RCADS-25, Revised Child Anxiety and Depression Scale 25; SDQ, Strengths and Difficulties Questionnaire.

In exploratory sex-stratified analysis, girls had higher scores than boys for all measures of mental health, and had lower well-being. We found statistical evidence for interactions between sex and ethnicity on all six measures. Visual inspection of sex-stratified scores suggested that the difference between white British and Pakistani adolescents was larger among girls than boys, and the difference may be reversed for eating-related problems such that Pakistani boys reported more eating-related problems than white British boys (a post hoc t-test gave p<0.001). Selected sex-stratified scores are presented in figure 4, with all outcomes presented in online supplemental file 1 section 6. We did not find evidence for interactions between sex and free school meal eligibility on any outcomes, and free school meal eligibility was associated with worse mental health and well-being within both boys and girls.

## Discussion

### Summary of findings

In this study of adolescents living in an urban and socioeconomically deprived UK setting, those of Pakistani ethnicity reported better mental health and well-being than white British adolescents. These differences were most pronounced for symptoms related to hyperactivity and anxiety. Consistent with previous evidence, lower family income as indicated by eligibility for free school meals was associated with poorer mental health and well-being across multiple measures.

### Comparison with existing evidence

Studies into the relationship between ethnicity and adolescent mental health have varied results. The mental health of minority groups is likely to depend on many factors including the specific outcome being studied, the age of participants, migration and colonial histories, cultural factors, deprivation, ethnic density^26^ and other forms of social capital. Consequently, quantitative studies in this field cannot easily be combined into a single summary estimate; rather, their findings must be interpreted within the relevant social and cultural context.

In the UK, most studies suggest that adolescents from minority ethnic groups have similar or better mental health and well-being than white British adolescents (the majority group) despite higher rates of poverty.^27–33^ Much of this evidence comes from general population studies dominated by white British participants. For example, data from the Millennium Cohort Study (82% white British) indicate that 14-year-olds of Pakistani and Bangladeshi ethnicities report fewer emotional and behavioural problems than their White British peers.^30^

Studies with larger proportions of minority ethnic adolescents include: (1) the REACH (Resilience, Ethnicity & AdolesCent Mental Health) study in London, where the largest ethnic group was black African, found similar mental health across ethnic groups despite higher deprivation and other risk factors in minority groups^31^; (2) a study in Leicester and two London suburbs with substantial Indian populations, in which adolescents of Indian ethnicity reported lower behavioural and emotional problems than white British adolescents^33^; (3) a study in east London, where adolescents of Bangladeshi ethnicity, the largest group, showed substantially lower risk of psychological distress compared with white British adolescents.^32^ However, some studies indicate poorer mental health among certain minority groups. For example, other results from REACH found that children of black Caribbean and mixed white and black ethnicities reported more conduct problems than other children.^34^

Household survey data in the USA also show mixed results. Some studies suggest that common mental health problems and major depressive episodes are less common among adolescents in minority racial groups than among white adolescents^35–37^; while other studies suggest that adolescents from minority racial groups are more likely to experience mental health problems.^38^

Evidence relating to mental health and socio-economic status is clearer, with deprivation and poverty consistently linked to poorer mental health among adolescents across both high-income and low-income countries.^39–52^ This association has persisted over several decades.^53^ Socio-economic inequalities in adolescent mental health are summarised in three international systematic reviews.^40,54,55^ These inequalities have been observed across multiple indicators of socio-economic position including parental income, education, occupation and neighbourhood deprivation, and across a wide range of mental health and well-being outcomes. Our results in relation to family income are consistent with this evidence.

Other research has identified mechanisms that could underlie these inequalities. These mechanisms include stress related to insufficient material resources, housing insecurity, parental factors such as limited parental availability and parenting styles,^39^ employment or caregiving responsibilities during adolescence, adverse experiences in childhood, intergenerational mechanisms in which parents with mental health problems are more likely to experience poverty; and the compounding effects of COVID-19 lockdowns on these mechanisms.^56–58^

### Relevance for policy, practice and future research

Fiscal policies that reduce poverty are likely to improve adolescent mental health. Given that most mental health problems start during this period of life, such policies are likely to have long-term benefits for quality of life, employment and social participation.

One possible reason why adolescents of Pakistani ethnicity report better mental health and well-being than adolescents of white British ethnicity is the centrality of community institutions and ‘bonding’ social capital in Pakistani communities which may provide resilience.^59^ ‘Bonding’ social capital refers to social networks within communities. In other evidence from Bradford, adolescents of Pakistani heritage typically have larger household sizes and more regular religious participation.^60^ Additionally, ethnicity may be associated with different parenting styles, with evidence from an earlier follow-up of this cohort suggesting that Pakistani mothers felt more confident about their abilities as a parent and may lose their temper less often than parents in other groups.^61^ Research covering four European countries suggested that a mental health advantage among adolescents with immigrant backgrounds is partly explained by family cohesion and other family factors.^29^

Religious or family institutions that may be beneficial in one group cannot be established in another through policy. However, a clearer understanding of these factors may inform more general policy addressing the current crisis in adolescent mental health. Some ethnic communities may have resilient institutions that support social participation among adolescents. The policy implications may relate to long-term investment in resilient social institutions that are relevant to adolescents, such as youth groups; sporting, artistic and cultural opportunities; and religious groups. Research into mediating factors that explain ethnic inequalities in adolescent mental health may also help guide policy.

### Strengths and limitations

This research has three main strengths. First, it is a study of contemporary adolescents, while many existing studies of adolescent mental health use older data in which adolescents were likely exposed to different risk factors. Second, Born in Bradford captures a large group of Pakistani ethnicity, allowing a powerful comparison with adolescents of white British ethnicity, in contrast with many studies that have small numbers in minority groups. For example, in a study of ethnic inequalities in the Millennium Cohort Study, the largest minority ethnic group is ‘mixed’, comprising 5% of the sample.^30^ As a result, many studies reporting mental health for subgroups such as people of Pakistani ethnicity find non-significant results due to small numbers in these subgroups.^17,62^ Third, we used a range of measures of mental health and well-being, while most other research focuses on one measure, predominantly SDQ,^17^ and often with dichotomised outcomes.^30^

The research has four main limitations. First, the results may have limited generalisability to other minority ethnic groups. Social capital or other factors that affect adolescent mental health vary across places. Similarly, the better mental health observed for adolescents of Pakistani ethnicity may reflect poor mental health in adolescents of white British ethnicity within Bradford, rather than an ‘advantage’ in the Pakistani group. Second, there may be cultural bias in the research instruments, such that observed ethnic differences could be partly explained (or underestimated) due to differences in interpretation of survey questions and stigma surrounding mental health. Lower levels of symptoms among adolescents of Pakistani ethnicity could therefore partly reflect ethnic differences in how psychological distress is understood and reported. Evidence from the UK and Norway suggests that the SDQ demonstrates acceptable measurement invariance across ethnic groups,^63,64^ while a multicountry study of the RCADS found that only a small minority of items exhibited differential item functioning and concluded that the measure has good cross-cultural validity.^65^ There has been less evaluation of cultural equivalence for other measures used in this study. Although cultural biases may contribute to some of the observed differences, the absence of strong evidence for substantial measurement non-equivalence in SDQ and RCADS, together with the consistency of findings across multiple constructs, suggests that the findings are unlikely to be explained solely by measurement artefact. Third, the construct ‘ethnicity’ is reductive in quantitative research to allow comparisons between groups, but people within one category are unlikely to have a uniform experience. Fourth, while we standardised effect sizes, Cohen’s *d* is relative to the distribution of the outcome and a larger effect size may not indicate a more important inequality when comparing different outcomes.

## Conclusion

Poverty is associated with worse adolescent mental health, and fiscal policies that reduce poverty are likely to improve outcomes. While adolescents of Pakistani ethnicity typically lived in more deprived neighbourhoods, they reported better mental health than white British adolescents, particularly in relation to emotional and behavioural problems. This may suggest that cultural factors related to ethnicity might affect adolescent mental health.

## Supplementary material

10.1136/bmjph-2025-004528 online supplemental file 1

10.1136/bmjph-2025-004528 online supplemental file 2

10.1136/bmjph-2025-004528 online supplemental file 3

## Data Availability

Researchers can apply to use data from Born in Bradford by completing an ‘Expression of Interest’ form. Following an approved application, we can share data under a Data Sharing Agreement. Guidance and the form are available here: https://borninbradford.nhs.uk/our-data/how-to-access-data/. Following our ethical approvals, we cannot make individual-level data publicly available.
