# Supplementary material for: Ethnic and income-related inequalities in mental health among adolescents: a cross-sectional study in Bradford, UK
Source: BMJ Public Health. 2026 Sep 15;4(3):e004528. doi: 10.1136/bmjph-2025-004528 (PMC13583858; doi:10.1136/bmjph-2025-004528)
Supplement: Supplementary file 1 [file bmjph-4-3-s001.pdf]

Supplementary information

1. Outcome measures and completeness of responses 2
2. Sample characteristics 3
3. Histograms of outcome measures, including subscales 4
4. Distribution of outcome measures, stratified by ethnicity and free school meal eligibility 5
5. Comparison between main results and complete case analysis 7
6. Exploratory sex-stratified results 8
7. STROBE checklist 10

1. Outcome measures and completeness of responses

Table: Outcome measures and completeness of responses

Instrument	Construct	Number of items	Range of scores	Academic years receiving the instrument	Number of participants eligible to respond	Completing the instrument Number of participants (%)			
						Instrument not done	Partial response	All items complete	At least one item
RCADS25	Anxiety and Depression	25	0-75	8 & 10	6,130	1,449 (23.6)	593 (9.7)	4,088 (66.7)	4,681 (76.4)
	Anxiety (subscale)	15	0-45		6,130	1,457 (23.8)	431 (7.0)	4,242 (69.2)	4,673 (76.2)
	Depression (subscale)	10	0-30		6,130	1,455 (23.7)	335 (5.5)	4,340 (70.8)	4,675 (76.3)
SWEMWBS	Mental wellbeing	7	7-35	8, 9, and 10	9,496	1,262 (13.3)	582 (6.1)	7,652 (80.6)	8,234 (86.7)
ULS4	Loneliness	4	4-12	8, 9, and 10	9,496	1,322 (13.9)	233 (2.5)	7,941 (83.6)	8,174 (86.1)
SDQ	Emotional and behavioural problems	20	0-40	9	3,366	663 (19.7)	279 (8.3)	2,424 (72.0)	2,703 (80.3)
	Emotional problems (subscale)	5	0-10		3,366	664 (19.7)	90 (2.7)	2,612 (77.6)	2,702 (80.3)
	Peer problems (subscale)	5	0-10		3,366	674 (20.0)	103 (3.1)	2,589 (76.9)	2,692 (80.0)
	Conduct problems (subscale)	5	0-10		3,366	669 (19.9)	110 (3.3)	2,587 (76.9)	2,697 (80.1)
	Hyperactivity (subscale)	5	0-10		3,366	664 (19.7)	116 (3.4)	2,586 (76.8)	2,702 (80.3)
EDE-QS	Eating disorders	11	0-36	8, 9, and 10	9,496	1,030 (10.8)	962 (10.1)	7,504 (79.0)	8,466 (89.2)
PLIKS	Unusual or psychosis-like symptoms	8	0-16	10	2,745	650 (23.7)	180 (6.6)	1,915 (69.8)	2,095 (76.3)

2. Sample characteristics

Table: Sample characteristics, compared to all adolescents in Bradford and England

Variable	Level	Study sample N (%) Median [IQR]	Participating schools N (%) Median [IQR]	Bradford % Median [IQR]	England % Median [IQR]
Total		9,496 (100.0)	13,044 (100.0)	100.0	100.0
Age at survey completion (years)		13.9 [13.2-14.6]	-	-	-
Season of survey completion	Autumn 2023	1,908 (20.1)	-	-	-
	Winter 2023/24	3,319 (35.0)	-	-	-
	Spring 2024	1,938 (20.4)	-	-	-
	Summer 2024	2,331 (24.5)	-	-	-
Sex (a)	Female	4,952 (52.1)	6,744 (51.7)	48.9	48.7
	Male	4,544 (47.9)	6,300 (48.3)	51.1	51.3
National curriculum year group (a)	8	3,366 (35.4)	4,633 (35.5)	33.4	34.0
	9	3,385 (35.6)	4,529 (34.7)	33.6	33.3
	10	2,745 (28.9)	3,882 (29.8)	33.0	32.7
Population density (residents/km2) (b)		4,984 [3,291-7,202]	4,923 [2,954-7,053]	4,806 [2,868-7,259]	1,407 [3,742-6,176]
Special Education Needs (a)	None	7,700 (81.1)	10,149 (77.8)	80.2	81.3
	SEN	1,225 (12.9)	1,960 (15.0)	14.9	13.4
	EHCP	137 (1.4)	346 (2.7)	4.9	5.4
	Missing	434 (4.6)	589 (4.5)	-	-
Eligible for free school meals (a)	No	6,737 (70.9)	8,767 (67.2)	66.5	73.5
	Yes	2,759 (29.1)	4,277 (32.8)	33.5	26.5
Ethnicity (a)	Asian and Asian British	4,589 (48.3)	5,878 (45.1)	44.2	13.3
	- *Of which British Pakistani*	3,885 (40.9)	5,008 (38.4)	37.3	4.6
	- *Of which Other Asian*	704 (7.4)	870 (6.7)	6.9	8.7
	White	3,705 (39.0)	5,503 (42.2)	43.1	68.9
	- *Of which White British*	3,271 (34.4)	4,855 (37.2)	37.7	61.4
	- *Of which Other White*	434 (4.6)	648 (5.0)	5.5	7.5
	Black and Black British	341 (3.6)	430 (3.3)	2.9	6.7
	Mixed ethnicities	498 (5.2)	731 (5.6)	5.8	6.7
	Other	171 (1.8)	214 (1.6)	1.8	2.4
	Missing	192 (2.0)	288 (2.2)	2.1	2.0
Index of Multiple deprivation 2019 quintile (b)	1 (most deprived)	5,888 (62.0)	8,033 (61.6)	57.1	23.7
	2	1,909 (20.1)	2,638 (20.2)	19.5	20.0
	3	656 (6.9)	1,001 (7.7)	9.4	18.5
	4	433 (4.6)	678 (5.2)	7.8	18.2
	5 (least deprived)	603 (6.4)	682 (5.2)	6.2	19.7
	Missing	7 (0.1)	12 (0.1)	-	-

a) Data for the comparison column are from the Department for Education 2023/24 school census for pupils in year 8-10 at state-funded secondary schools in England, accessed via https://explore-education-statistics.service.gov.uk/.

b) Population density and Index of Multiple Deprivation of home address are calculated from ONS data for 12-15 year-olds in England, using data on population by single-year-of-age, land area, and Index of Multiple Deprivation, at Lower Level Super Output Area level.

3. Histograms of outcome measures, including subscales

Figure: Histograms of all measures included in the study, including subscales

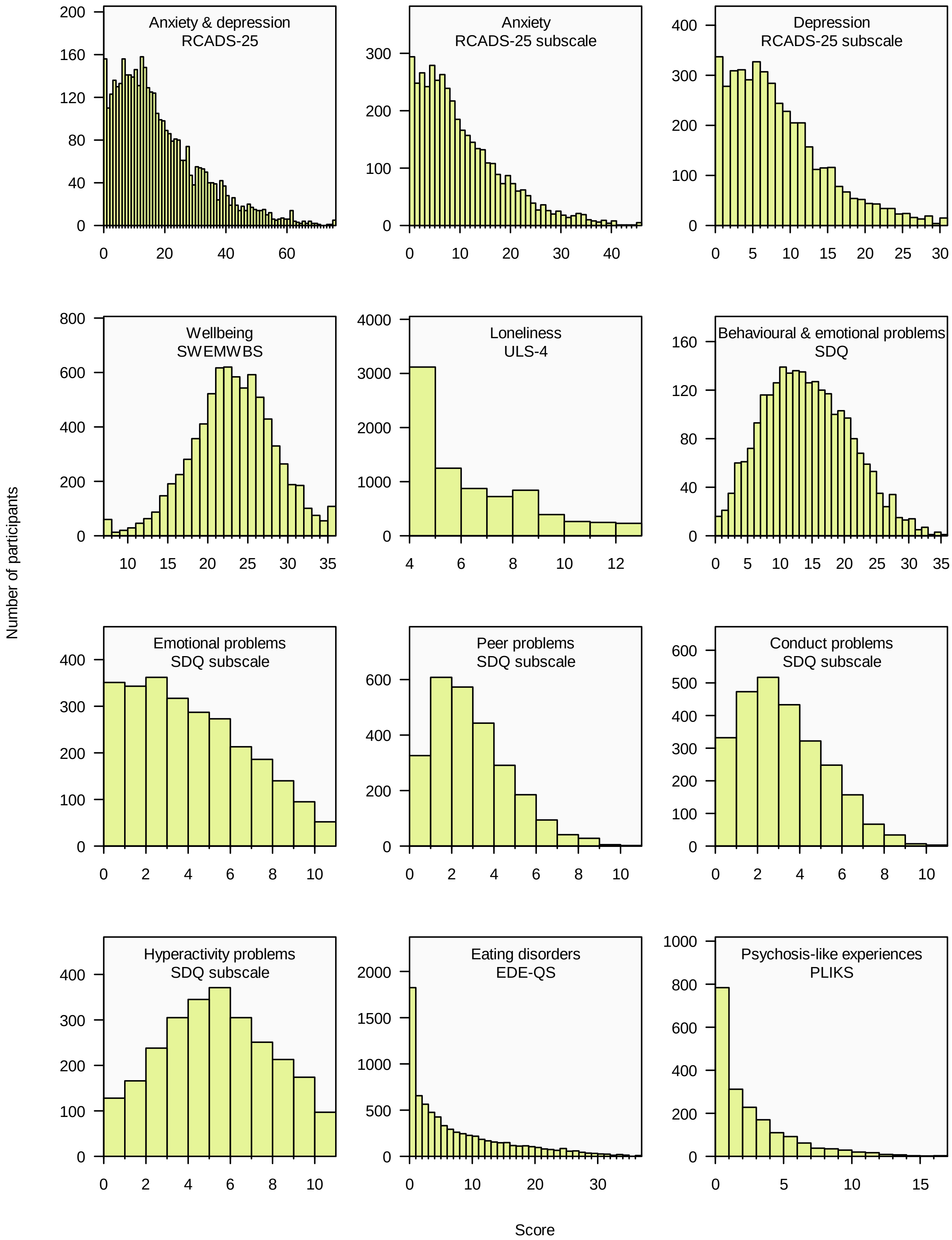

4. Distribution of outcome measures, stratified by ethnicity and free school meal eligibility

Figure: empirical cumulative distributions of outcomes, stratified by ethnicity (White British and Pakistani participants only) - red = British Pakistani, blue = White British. Red and blue numbers below the x-axis indicate lower quartile, median, and upper quartile.

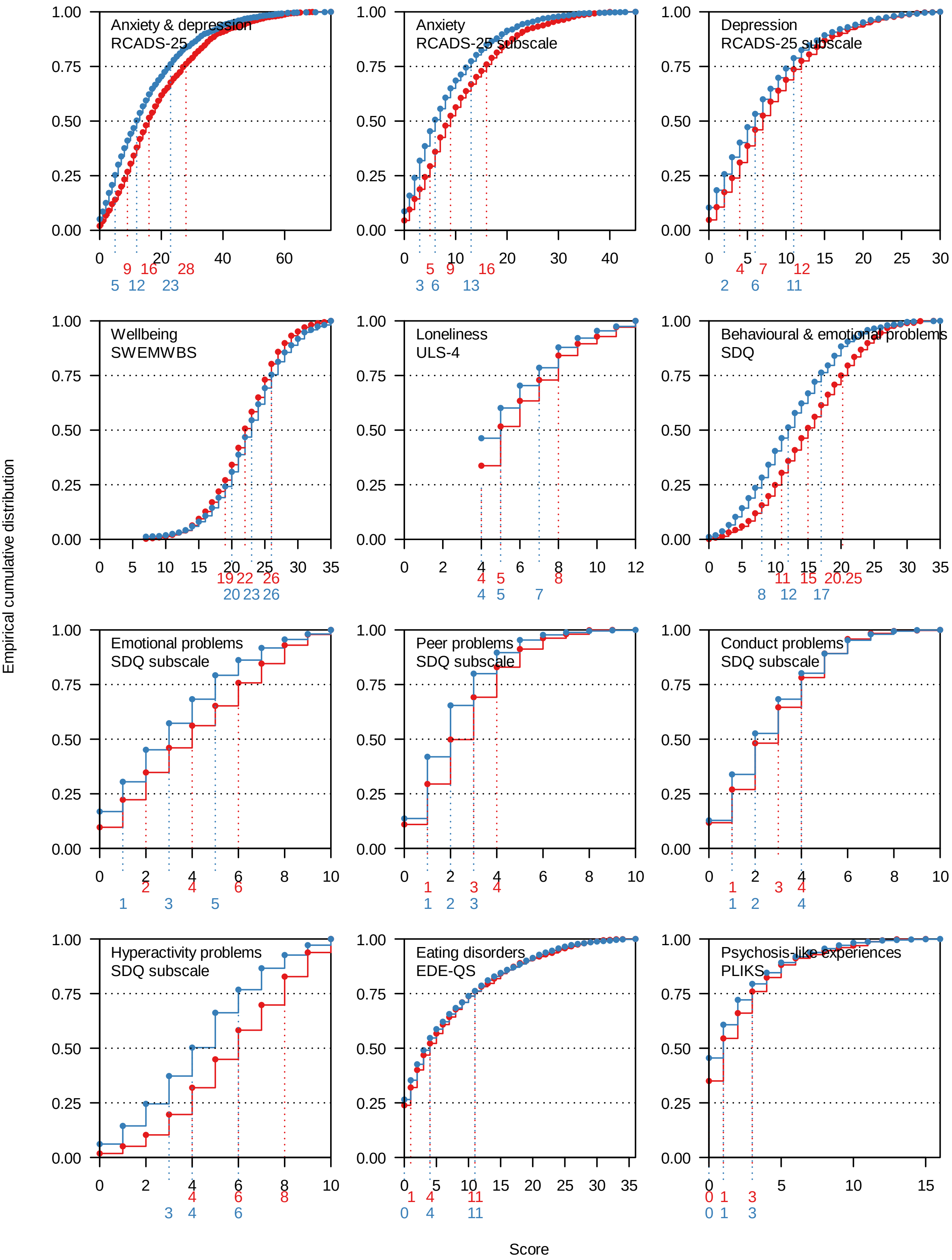

Figure: empirical cumulative distributions of outcomes, stratified by eligibility for free school meals, red = not eligible, blue = eligible

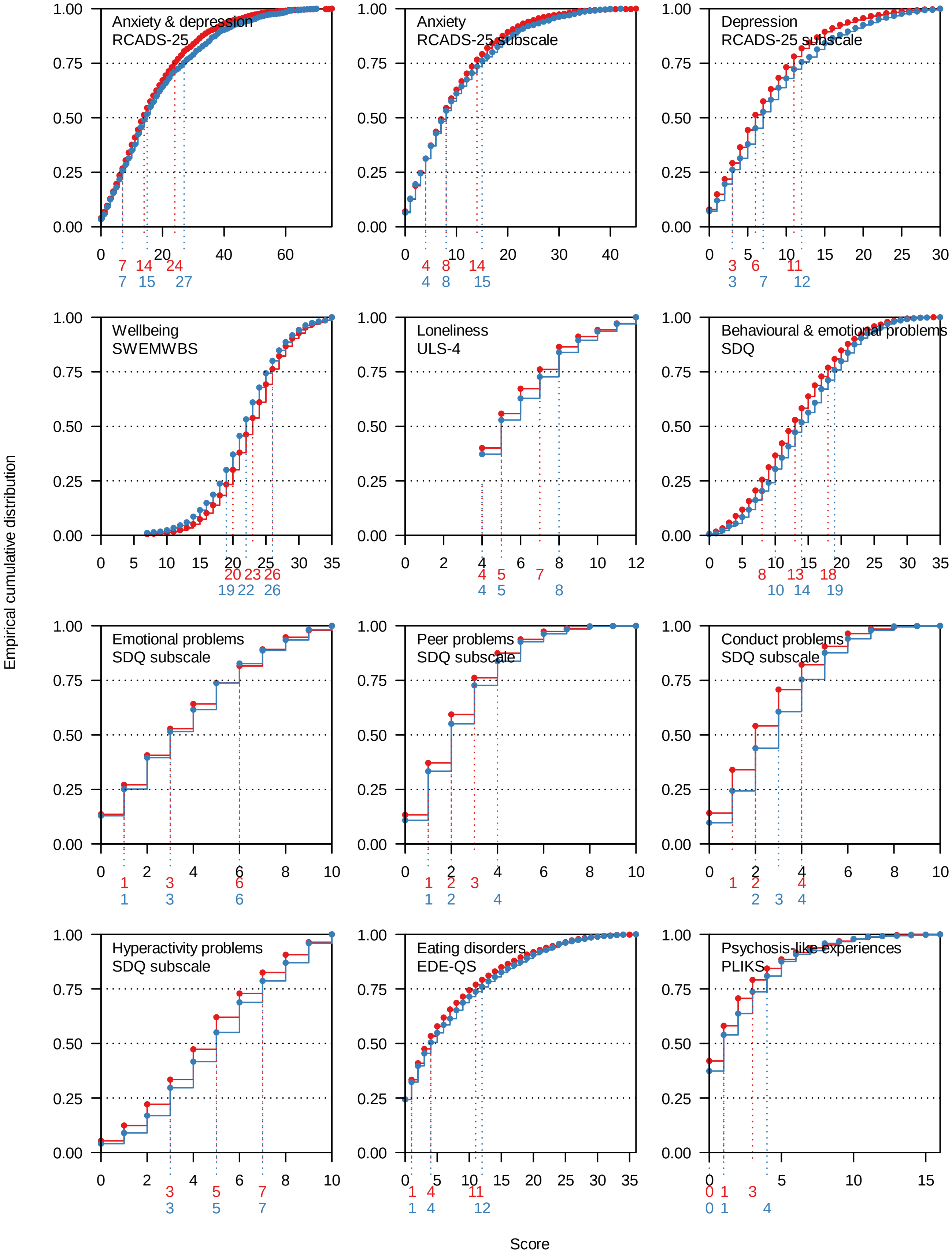

5. Comparison between main results and complete case analysis

Figure: Comparison between main results (using multiple imputation) and a complete case analysis. The complete case analysis is shown with dashed confidence bars.

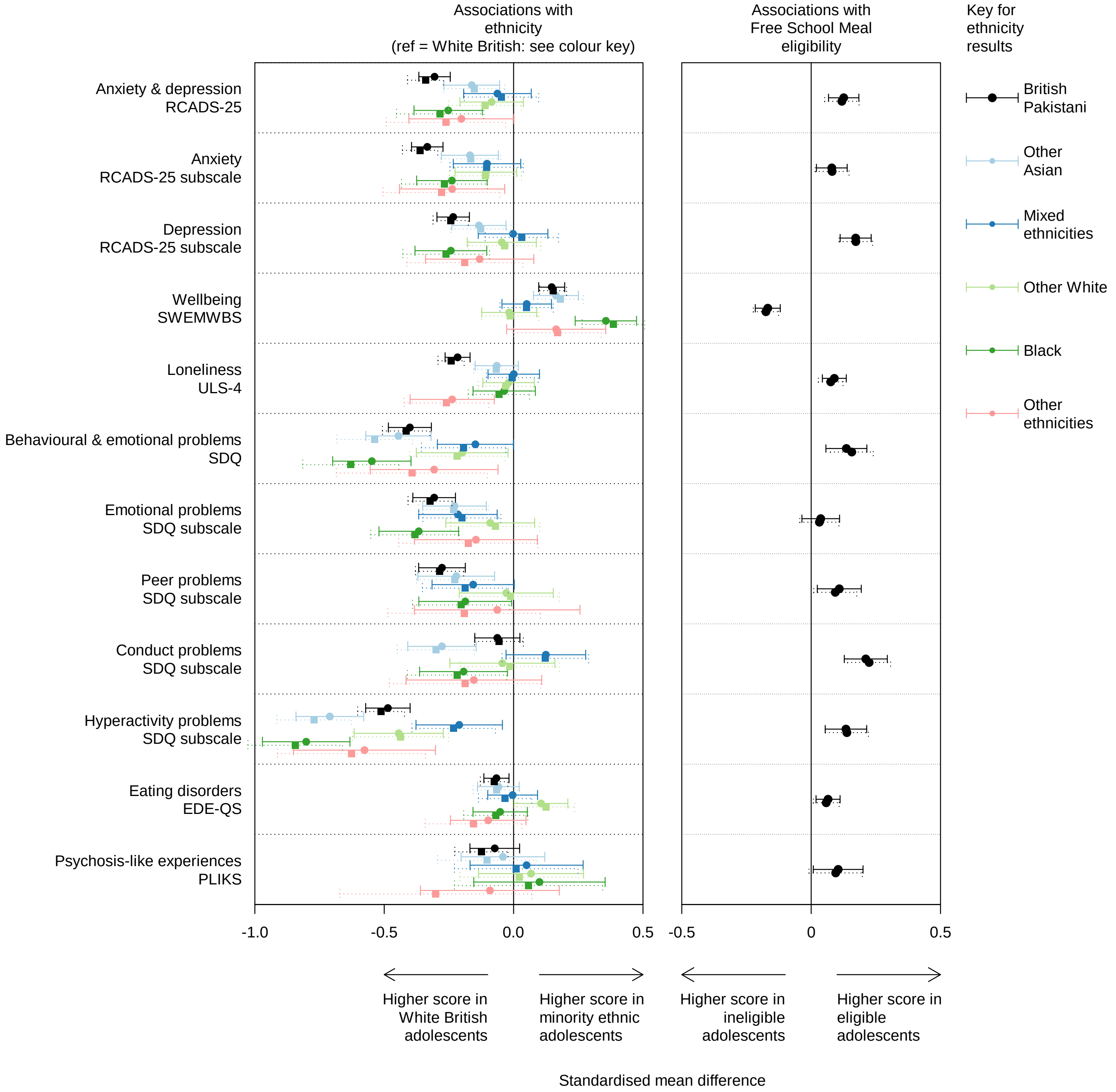

6. Exploratory sex-stratified results

Figure: Outcome scores stratified by ethnicity and sex. Scores are predicted from regression models including an interaction term between ethnicity and sex (95% CIs). P-values are based on a likelihood ratio test between models with and without the interaction term.

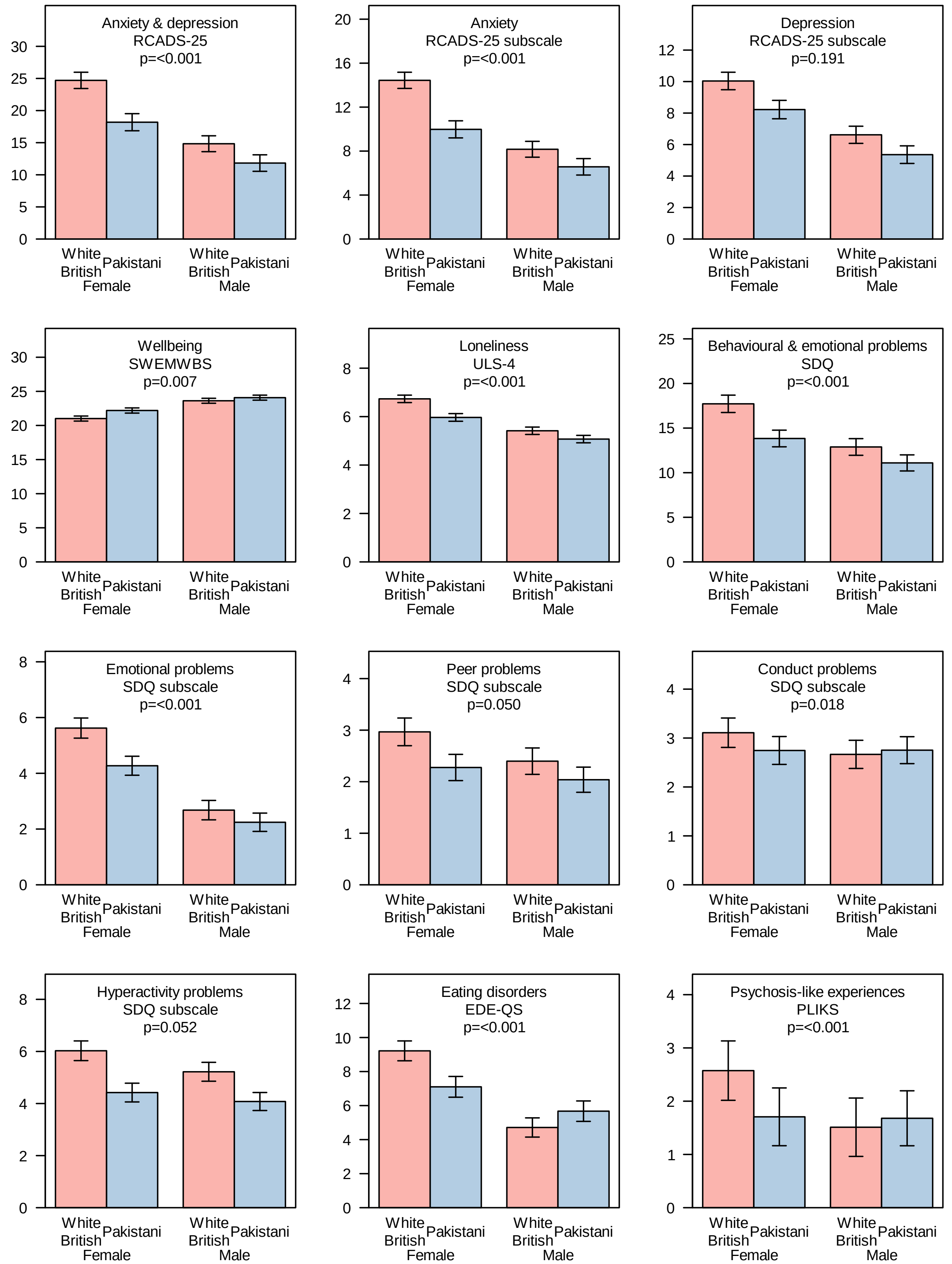

Figure [x]: Outcome scores stratified by free school meal eligibility and sex. Scores are predicted from regression models including an interaction term between eligibility and sex (95% CIs). P-values are based on a likelihood ratio test between models with and without the interaction term.

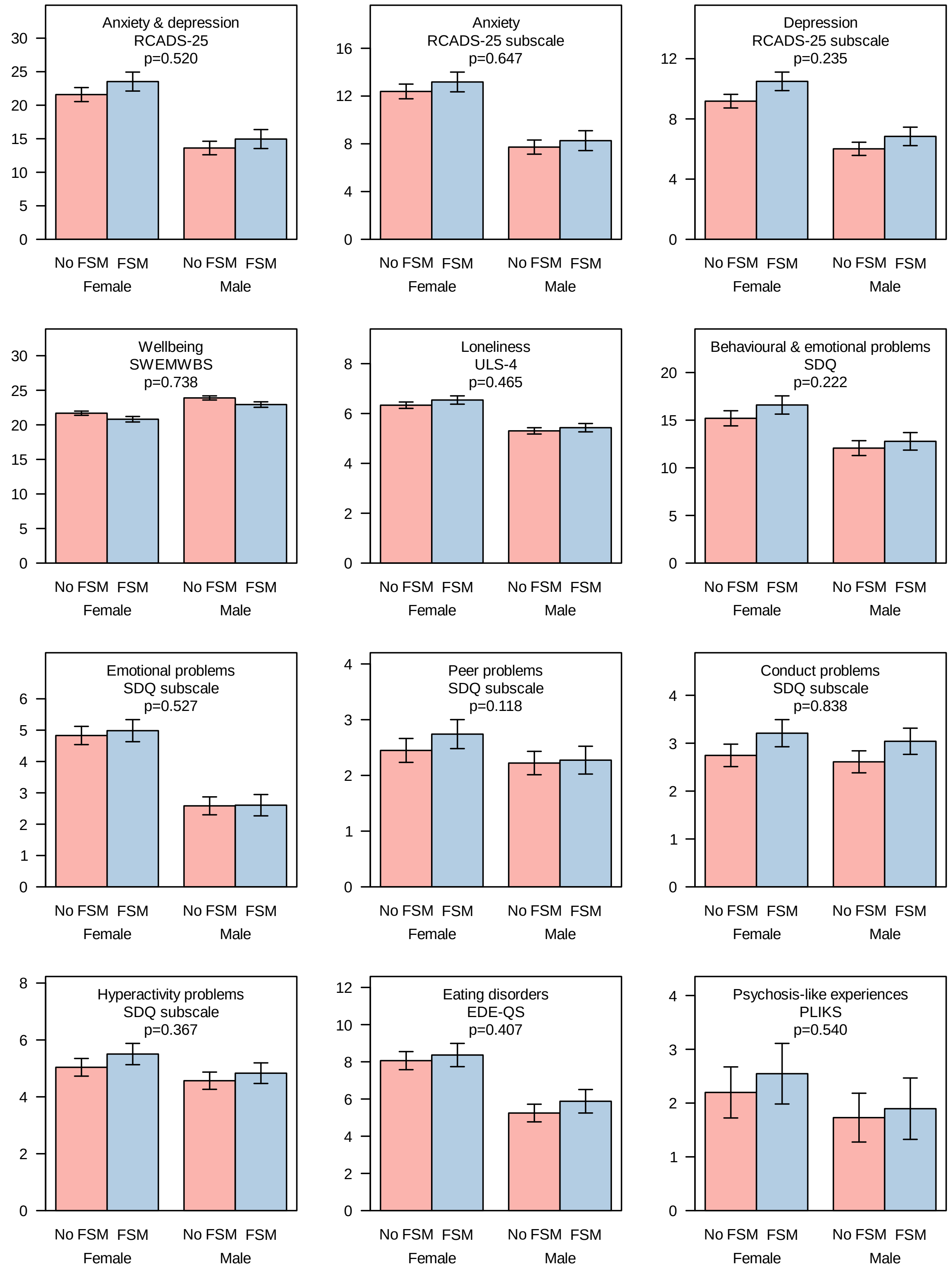

7. STROBE checklist

Section	Item No	Recommendation	Section
Title and abstract	1	(*a*) Indicate the study's design with a commonly used term in the title or the abstract	Title
		(*b*) Provide in the abstract an informative and balanced summary of what was done and what was found	Abstract
Introduction			
Background/ rationale	2	Explain the scientific background and rationale for the investigation being reported	Abstract/background; Introduction
Objectives	3	State specific objectives, including any prespecified hypotheses	N/A (descriptive study)
Methods			
Study design	4	Present key elements of study design early in the paper	Methods/design; Methods/data source; Methods/outcomes; Methods/exposures; Methods;statistical analysis
Setting	5	Describe the setting, locations, and relevant dates, including periods of recruitment, exposure, follow-up, and data collection	Methods/data source and participants
Participants	6	(*a*) Give the eligibility criteria, and the sources and methods of selection of participants	Methods/data source and participants; Recruitment flow chart
Variables	7	Clearly define all outcomes, exposures, predictors, potential confounders, and effect modifiers. Give diagnostic criteria, if applicable	Methods/outcomes; Methods/exposures; Methods/other variables
Data sources/ measurement	8*	For each variable of interest, give sources of data and details of methods of assessment (measurement). Describe comparability of assessment methods if there is more than one group	Methods/outcomes; Methods/exposures; Methods/other variables
Bias	9	Describe any efforts to address potential sources of bias	Methods/statistical analysis
Study size	10	Explain how the study size was arrived at	Recruitment flow chart
Quantitative variables	11	Explain how quantitative variables were handled in the analyses. If applicable, describe which groupings were chosen and why	Methods/outcomes; Methods/exposures; Methods/other variables
Statistical methods	12	(*a*) Describe all statistical methods, including those used to control for confounding	Methods/statistical analysis
		(*b*) Describe any methods used to examine subgroups and interactions	Methods/statistical analysis
		(*c*) Explain how missing data were addressed	Methods/statistical analysis
		(*d*) If applicable, describe analytical methods taking account of sampling strategy	N/A
		(*e*) Describe any sensitivity analyses	N/A
Results			

Section	Item No	Recommendation	Section
Participants	13*	(a) Report numbers of individuals at each stage of study—eg numbers potentially eligible, examined for eligibility, confirmed eligible, included in the study, completing follow-up, and analysed	Recruitment flow chart
		(b) Give reasons for non-participation at each stage	Recruitment flow chart
		(c) Consider use of a flow diagram	Recruitment flow chart
Descriptive data	14*	(a) Give characteristics of study participants (eg demographic, clinical, social) and information on exposures and potential confounders	Results/Table 2; Supplementary Information
		(b) Indicate number of participants with missing data for each variable of interest	Methods/Table 1
Outcome data	15*	Report numbers of outcome events or summary measures	Results/Figure 2 (histograms)
Main results	16	(*a*) Give unadjusted estimates and, if applicable, confounder-adjusted estimates and their precision (eg, 95% confidence interval). Make clear which confounders were adjusted for and why they were included	Results; Results/Figure 3; Results/Table 4
		(*b*) Report category boundaries when continuous variables were categorized	N/A
		(*c*) If relevant, consider translating estimates of relative risk into absolute risk for a meaningful time period	N/A
Other analyses	17	Report other analyses done—eg analyses of subgroups and interactions, and sensitivity analyses	Results; Results/Figure 4
Discussion			
Key results	18	Summarise key results with reference to study objectives	Discussion/Summary of findings
Limitations	19	Discuss limitations of the study, taking into account sources of potential bias or imprecision. Discuss both direction and magnitude of any potential bias	Discussion/Strengths and limitations
Interpretation	20	Give a cautious overall interpretation of results considering objectives, limitations, multiplicity of analyses, results from similar studies, and other relevant evidence	Discussion/Relevance for policy, practice, and future research
Generalisability	21	Discuss the generalisability (external validity) of the study results	Discussion/Strengths and limitations
Other information			
Funding	22	Give the source of funding and the role of the funders for the present study and, if applicable, for the original study on which the present article is based	Methods/Role of the funding source; Additional information/funding
