# Supplementary material for: Ethnic and income-related inequalities in mental health among adolescents: a cross-sectional study in Bradford, UK
Source: BMJ Public Health. 2026 Sep 15;4(3):e004528. doi: 10.1136/bmjph-2025-004528 (PMC13583858; doi:10.1136/bmjph-2025-004528)
Supplement: Supplementary file 2 [file bmjph-4-3-s002.pdf]

Bradford Teaching Hospitals
NHS Foundation Trust

BiB Age of Wonder

MODULE ONE (231)
2023-24

Socioeconomics

Arts and Culture

Physical Health

Health Behaviours

01274 274474
ageofwonder@bthft.nhs.uk
www.borninbradford.nhs.uk

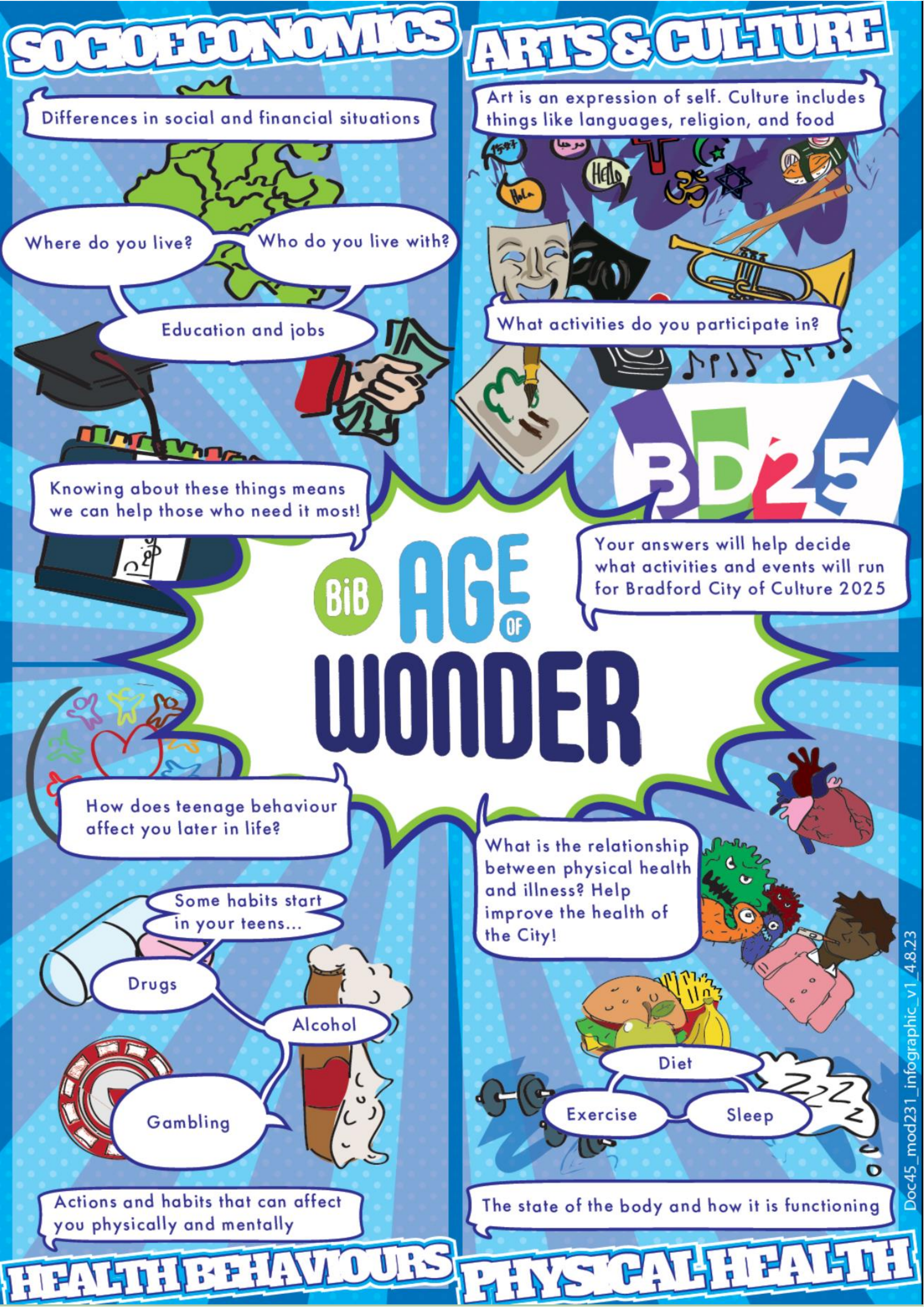

SOCIOECONOMICS
Differences in social and financial situations
Where do you live?
Who do you live with?
Education and jobs
Knowing about these things means we can help those who need it most!
ARTS & CULTURE
Art is an expression of self. Culture includes things like languages, religion, and food
Hello
Hola
What activities do you participate in?
BD25
Your answers will help decide what activities and events will run for Bradford City of Culture 2025
BiB
AGE OF WONDER
How does teenage behaviour affect you later in life?
Some habits start in your teens...
Drugs
Alcohol
Gambling
Actions and habits that can affect you physically and mentally
HEALTH BEHAVIOURS
What is the relationship between physical health and illness? Help improve the health of the City!
Diet
Exercise
Sleep
The state of the body and how it is functioning
PHYSICAL HEALTH
Doc45_mod231_infographic_v1_4.8.23

CONTENTS 1

SECTION 1 - DEMOGRAPHICS

Ethnicity

Languages

Religion

Sex and Gender Identity

Disability

SECTION 2 – SOCIOECONOMICS

Material Possessions

Household Structure

Family

Your Financial Resources

Food Availability

Social Comparison

Neighbourhood

SECTION 3 – ARTS AND CULTURE

Activities

Pride in Place

01274 274474

ageofwonder@bthft.nhs.uk

www.borninbradford.nhs.uk

CONTENTS 2

SECTION 4 – PHYSICAL HEALTH I

General Health

Hearing and Sight

Food and Diet

Eating Habits

Oral Health

Puberty

SECTION 5 – HEALTH BEHAVIOURS

Smoking and Vaping

Alcohol

Drugs

Gambling

Knives

SECTION 6 – PHYSICAL HEALTH II

Physical Activity

Sedentary Activity

Sleep

01274 274474
ageofwonder@bthft.nhs.uk
www.borninbradford.nhs.uk

BiB AGE OF WONDER

Bradford Teaching Hospitals
NHS Foundation Trust

Item No.	Items	Response format		
1	Participant Study ID	Text		
2	Year Group	8	9	10

SECTION 1 – DEMOGRAPHICS

In this section we ask about things like your ethnicity, language, and religion. This helps us understand young people's answers from different backgrounds. Remember, if you do not want to answer a question, you can skip it.

Item No.	Items	Response format
3	What is the first letter of your surname?	Drop down A-Z
4	What day of the month is your birthday?	Drop down 1-31
5	What is your country of birth?	Drop down 1-200
6	**IF NON-UK COUNTRY SELECTED FOR Q6**<br>How many years have you lived in the UK?	Drop down 1-15

Ethnicity

Ethnicity is the racial and/or cultural background you associate with (e.g., White-English, Asian-Pakistani) Choose one option then choose one box below to best describe your ethnic group or background.

Item No.	Items	Response format
7	What is your ethnicity?	White<br>Mixed or Multiple Ethnic Groups<br>Asian or Asian British<br>Black, Black British, Carribean or African<br>Arab<br>Other Ethnic Groups

01274 274474
ageofwonder@bthft.nhs.uk
www.borninbradford.nhs.uk

8	**IF SELECTED WHITE TO Q7**<br>Tick one box to best describe your ethnic group or background.	English, Welsh, Scottish, Northern Irish, British<br>Irish<br>Gypsy or Irish Traveller<br>Roma<br>Polish<br>Slovakian<br>Romanian<br>Czech<br>Any other White background (please specify)
9	**IF SELECTED ANY OTHER WHITE BACKGROUND TO Q8**<br>Please specify.	Text box
10	**IF SELECTED MIXED OR MULTIPLE ETHNIC GROUPS TO Q7**<br>Tick one box to best describe your ethnic group or background.	White and Black Carribean<br>White and Black African<br>White and Asian<br>Any other mixed or multiple ethnic background (please specify)
11	**IF SELECTED ANY OTHER MIXED OR MULTIPLE ETHNIC BACKGROUND TO Q10**<br>Please specify.	Text box
12	**IF SELECTED ASIAN OR ASIAN BRITISH TO Q7**<br>Tick one box to best describe your ethnic group or background.	Indian<br>Pakistani<br>Bangladeshi<br>Chinese<br>Any other Asian background (please specify)
13	**IF SELECTED ANY OTHER ASIAN BACKGROUND TO Q12**<br>Please specify.	Text box
14	**IF SELECTED BLACK, BLACK BRITISH, CARRIBEAN OR AFRICAN TO Q5**<br>Tick one box to best describe your ethnic group or background.	Carribean<br>African background (please specify)<br>Any other Black, Black British or Carribean background (please specify)
15	**IF SELECTED AFRICAN BACKGROUND TO Q14**<br>Please specify.	Text box

01274 274474
ageofwonder@bthft.nhs.uk
www.borninbradford.nhs.uk

16	**IF SELECTED ANY OTHER BLACK, BLACK BRITISH OR CARRIBEAN BACKGROUND TO Q14**<br>Please specify.	Text box
17	**IF SELECTED ARAB TO Q7**<br>Tick one box to best describe your ethnic group or background.	African Arab<br>Middle-Eastern Arab
18	**IF SELECTED ANY OTHER ETHNIC GROUP TO Q7**<br>Please specify.	Text box

Languages

Item No.	Items	Response format
19	Which of these languages is usually spoken at home? Select all that apply.	English<br>Urdu<br>Punjabi<br>Gujarati<br>Bengali<br>Hindko<br>Polish<br>Pashto<br>Other (please specify)
20	**IF OTHER SELECTED FOR Q19**<br>Please specify	Text box

Religion

Item No.	Items	Response format	
21	Do you consider yourself to have a religion?	YES	NO

22	What is your religion?	Christianity (including Church of England, Catholic, Protestant, and all other Christian denominations)<br>Budhism<br>Hinduism<br>Judaism<br>Islam<br>Sikhism<br>Any other Religion (please specify)
23	**IF SELECTED ANY OTHER RELIGION FOR Q22**<br>Please specify	Text box

Sex and Gender identity

The next questions are about sex and gender identity. In order for our research to be compared with other studies we have used standard measures similar to the National Census. This information is used to monitor equality between groups of people of different genders and sexes. Equality monitoring helps make sure that everyone is treated fairly and helps identify what services are needed. You can choose not to answer any of the questions.

If you feel that there is not an answer that applies to you, there will be a space for you to enter your own response.

Item No.	Items	Response format
24	What is your sex? (The biological sex on your birth certificate. A question on gender identity will follow in the survey.)	Female<br>Male<br>Do not wish to answer<br>Wish to enter own response
25	**IF WISH TO ENTER OWN RESPONSE WAS SELECTED TO Q24**<br>Space to self-describe	Text box
26	What is your gender? (How you would describe yourself.)	Female<br>Male<br>Non-binary<br>Wish to enter own response<br>Do not wish to answer
27	**IF WISH TO ENTER OWN RESPONSE SELECTED TO Q26**<br>Space to self-describe	Text box

Disability

In this section of the survey, you will be asked questions about any disabilities you may have. Remember, if you do not want to answer a question you can skip it.

Item No.	Items	Response format	
28	Do you have any physical or mental health conditions or illnesses?	YES	NO
29	**ONLY IF SELECTED YES TO Q28**<br>Has this lasted, or is it expected to last, for 12 months or more?	YES	NO
30	**ONLY IF SELECTED YES TO Q28**<br>Do any of your illnesses or conditions reduce your ability to carry out day-to-day activities?	1. Yes, a lot<br>2. Yes, a little<br>3. Not at all	

SECTION 2 - SOCIOECONOMICS

Material Possessions

In this part of the survey, you will be asked questions about the things you have. Remember, if you do not want to answer a question, you can skip it.

Item No.	Items	Response format	
	**Which of these things do you have?**		
31	Your own mobile phone	YES	NO
32	A computer, laptop or tablet with internet at home	YES	NO
33	At least one family holiday away from home in a typical year	YES	NO
34	A family car, van or truck	YES	NO
35	A bedroom for yourself	YES	NO

Age of Wonder

Bradford Teaching Hospitals
NHS Foundation Trust

36	Compared to your friends, would you say your family is richer, about the same, poorer, or don't know?	1. Richer<br>2. Poorer<br>3. About the same<br>4. Don't know	
37	How often do you worry about how much money your family has?	1. Never<br>2. Some of the time<br>3. All of the time	
38	When you are at home on a typical day in Winter, are you (and everyone in your household) warm enough?	1. Yes – always<br>2. Yes – sometimes<br>3. No – rarely<br>4. No – never	
39	My parents/guardians have talked about the need to cut back on certain things to save money.	YES	NO
40	**ONLY IF SELECTED YES TO Q39**<br>Which of the following have your parents/guardians discussed cutting back on? (tick all that apply)	1. Using too much energy (e.g., gas or electric)<br>2. Luxuries (e.g., takeaways, eating out, new cars, new clothes, etc…)<br>3. Holidays and leisure activities (e.g., hobbies outside of school, trips etc…)<br>4. Car journeys to save fuel	

Household Structure

In this part of the survey, you will be asked questions about who you live with. All of your responses are completely confidential. Remember, if you do not want to answer a question you can skip it.

Item No.	Items	Response format
41	Where do you live most of the time?	1. I live with my parent(s)/carer(s)<br>2. I live at both of my parents houses, some of the time at each<br>3. I live with other family members<br>4. I live somewhere else

42	Who else lives in your home? If you live at both your parents' houses, some of the time each, just think about the house where you spend most of your time.	1. Mother<br>2. Father<br>3. Guardian<br>4. Step mother<br>5. Step father<br>6. Mother's partner<br>7. Father's partner<br>8. Siblings<br>9. Auntie<br>10. Uncle<br>11. Grandmother<br>12. Grandfather<br>13. Cousins<br>14. Other
43	**ONLY IF SELECTED 13 TO Q42**<br>Please specify	Text box

Family

Item No.	Items	Response format
44	Select your birth order. I am…	Drop down 1st -10th :
45	How often does your family get along together?	1. Never<br>2. Some of the time<br>3. Always
46	How often do you get along with your brothers, sisters, and other young people you live with?	1. Never<br>2. Some of the time<br>3. Always<br>4. I don't live with any siblings

Your Financial Resources

In this part of the survey, you will be asked questions about your own money. Remember, all of your responses are completely confidential. If you do not want to answer a question, you can skip it.

Item No.	Items	Response format	
	**Do you get money to spend on yourself from any of the following sources?**		
47	Regular pocket money or allowance	YES	NO
48	Money from doing chores or babysitting for family or relatives	YES	NO
49	Money from working in the family business	YES	NO
50	Money from a paid job	YES	NO
51	Given money by parents when I need it	YES	NO

Food Availability

In this part of the survey, you will be asked questions about the availability of food to you. Your answers will help local organisations support peoples' access to food in your community and across Bradford. Remember, all of your responses are completely confidential. If you do not want to answer a question you can skip it.

Item No.	Items	Response format	
52	We can't get the food we want because there is not enough money	YES	NO
53	I worry about not having enough to eat	YES	NO
54	I worry about how hard it is for my parents to get enough food for us	YES	NO
55	I feel hungry because there is not enough food to eat	YES	NO
56	I try not to eat a lot so that our food will last	YES	NO

Social Comparison

In this part of the survey, you will be asked questions about how you feel about comparisons people make between each other. Remember, all of your responses are completely confidential, if you do not want to answer a question you can skip it.

Item No.	Items	Response format
57	How often do you think that people compare you to others to see if you match up?	1. Never<br>2. Rarely<br>3. Sometimes<br>4. Mostly<br>5. Always

Neighbourhood

This part of the survey will ask you questions about your neighbourhood. Remember, if you do not want to answer a question you can skip it.

Item No.	Items	Response format
	**How do you rate the following where you live?**	
58	Your safety when going out after dark	1. Very poor<br>2. Poor<br>3. Ok<br>4. Good<br>5. Very good
59	Your safety when going out during the day	1. Very poor<br>2. Poor<br>3. Ok<br>4. Good<br>5. Very good
60	Your safety when going to and from school	1. Very poor<br>2. Poor<br>3. Ok<br>4. Good<br>5. Very good
61	In the last 12 months, have you been the victim of violence or aggression in the area where you live?	1. No<br>2. Not sure<br>3. Yes

AGE OF WONDER

Bradford Teaching Hospitals
NHS Foundation Trust

SECTION 3 – ARTS AND CULTURE

Activities

In this section we'll be asking all about your involvement in arts and culture activities. Your answers will help local organisations identify which arts and culture activities are most relevant to young people to engage in these activities.

Item No.	Items	Response format
	**Did you do any of the following activities in the last month?**	
62	Sing, play an instrument, or make music (either on your own or as part of a choir/band/orchestra)	1. No<br>2. Yes – once<br>3. Yes – more than once
63	Creative writing (outside of school or work)	1. No<br>2. Yes – once<br>3. Yes – more than once
64	Voluntary, charity or community work	1. No<br>2. Yes – once<br>3. Yes – more than once
65	Make your own drawing, painting, sculpture, or other artwork (things that are made by hand)	1. No<br>2. Yes – once<br>3. Yes – more than once
66	Make your own graphic designs, photographs, or films/videos (things that are made using digital technology, including content for social media like Instagram and TikTok)	1. No<br>2. Yes – once<br>3. Yes – more than once
67	Read for enjoyment	1. No<br>2. Yes – once<br>3. Yes – more than once
68	Participate in a poetry/spoken word session	1. No<br>2. Yes – once<br>3. Yes – more than once
69	Take part in theatre, dance, circus, or other performance activities	1. No<br>2. Yes – once<br>3. Yes – more than once

Item No.	Items	Response format
	**Did you attend any of the following events in the last month?**	
70	A party, dance, house party or nightclub.	1. No<br>2. Yes – once<br>3. Yes – more than once
71	Live sport (for example at a stadium).	1. No<br>2. Yes – once<br>3. Yes – more than once
72	A live music concert or gig.	1. No<br>2. Yes – once<br>3. Yes – more than once
73	Some other type of performance, such as a play, pantomime, opera, dance, or circus.	1. No<br>2. Yes – once<br>3. Yes – more than once
74	Youth clubs.	1. No<br>2. Yes – once<br>3. Yes – more than once
75	Scounts/ Guides/ Explorer scounts/ Rangers.	1. No<br>2. Yes – once<br>3. Yes – more than once
76	A library.	1. No<br>2. Yes – once<br>3. Yes – more than once
77	Museums or galleries, a historical place, or stately home.	1. No<br>2. Yes – once<br>3. Yes – more than once
78	A political meeting, march, rally or demonstration.	1. No<br>2. Yes – once<br>3. Yes – more than once

01274 274474
ageofwonder@bthft.nhs.uk
www.borninbradford.nhs.uk

79	A religious service or event.	1. No<br>2. Yes – once<br>3. Yes – more than once
80	Video gaming event or festival.	1. No<br>2. Yes – once<br>3. Yes – more than once

Pride in Place

In this section we'll be asking how you feel about your local area. By local area we mean your neighbourhood and surrounding areas. For example, the area(s) of Bradford in which you live and attend school.

Item No.	Items	Response format
	**How much do you agree or disagree with the following?**	
81	I am proud to live in my local area	1. Strongly agree<br>2. Agree<br>3. Neither agree nor disagree<br>4. Disagree<br>5. Strongly disagree<br>6. Don't know
82	Arts and cultural events and activities available in my local area make me feel proud of my local area	1. Strongly agree<br>2. Agree<br>3. Neither agree nor disagree<br>4. Disagree<br>5. Strongly disagree<br>6. Don't know
83	Arts and cultural events and activities available in my local area are of interest to me	1. Strongly agree<br>2. Agree<br>3. Neither agree nor disagree<br>4. Disagree<br>5. Strongly disagree<br>6. Don't know

Section 4 – PHYSICAL HEALTH I

General Health

This part of the survey will ask you questions about your health (e.g., your sleep, what you eat, your physical activity). Your answers in this section will help doctors, nurses and local charities understand how best to support good general health across Bradford. Remember, if you do not want to answer a question, you can skip it.

Item No.	Items	Response format
84	How good is your health in general?	1. Very good<br>2. Good<br>3. Fair<br>4. Bad<br>5. Very bad

Hearing and Sight

Item No.	Items	Response format	
85	Have you been told to, or do you need to wear glasses in order to see clearly?	YES	NO
86	**ONLY IF NO SELECTED FOR Q85**<br>Do you struggle to see distant items (e.g. the board in class), or near items (e.g. when reading a book) clearly?	1. No<br>2. Yes – Distant<br>3. Yes – Near	
87	**ONLY IF YES FOR Q85**<br>What is the reason you need to wear glasses or contact lenses?	1. I struggle to see things in the distance or far away clearly without glasses or contact lenses<br>2. I struggle to see things close to me clearly without glasses or contact lenses<br>3. A 'turn in an eye' or a 'lazy eye' since childhood<br>4. Other reason<br>5. Don't know	
88	**ONLY IF YES SELECTED FOR Q85**<br>At what age did you first wear glasses or contact lenses?	Choose 0-16 years	
89	Do you have any difficulty hearing or use a hearing aid, including if you cannot hear at all?	YES	NO

Food and Diet

Item No.	Items	Response format
90	How often do you eat breakfast over a week?	1. Every day<br>2. Most days<br>3. About once a week<br>4. Less often<br>5. Never
91	How often do you eat at least 2 portions of fruit per day?	1. Every day<br>2. Most days<br>3. About once a week<br>4. Less often<br>5. Never
92	How often do you eat at least 2 portions of vegetables per day?	1. Every day<br>2. Most days<br>3. About once a week<br>4. Less often<br>5. Never
93	How often do you drink diet drinks or sugar free drinks like diet cola, pepsi max, or sugar-free squash?	1. Every day<br>2. Most days<br>3. About once a week<br>4. Less often<br>5. Never
94	How often do you drink sugary drinks like regular cola or squash?	1. Every day<br>2. Most days<br>3. About once a week<br>4. Less often<br>5. Never
95	How often do you drink energy drinks or high caffeine drinks (e.g., Red Bull, Prime Energy, Monster Energy)	1. Every day<br>2. Most days<br>3. About once a week<br>4. Less often<br>5. Never
96	How often do you eat fast food such as McDonalds, Burger King, KFC or other fast food like that?	1. Every day<br>2. Most days<br>3. About once a week<br>4. Less often<br>5. Never

01274 274474
ageofwonder@bthft.nhs.uk
www.borninbradford.nhs.uk

Eating Habits

VALIDATED MEASURE: Eating Disorder Examination Questionnaire – Short Version (EDEQ-S)

The next questions will ask you about food and whether you are happy with your size and the way you look. Some people can get quite worried about these things, but may find it difficult to speak about them, whereas others will not worry about them at all. It is incredibly valuable to us if you can answer these questions even if this is something that does not give you any concern. It will help us understand what type of support is needed for teenagers, and help local services and organisations to improve things for young people.

Please remember, all of your responses are completely confidential and if you really do not want to answer a question, you can skip it. If anything upsets you, please talk to your teacher or access the information provided at the end of the survey.

Item No.	Items	Response format
	**On how many of the past 7 days...**	
97	Have you been deliberately trying to limit the amount of food you eat to influence your weight or shape (whether or not you succeeded)?	1. 0 days<br>2. 1-2 days<br>3. 3-5 days<br>4. 6-7 days
98	Have you gone for long periods of time (e.g., 8 or more waking hours) without eating anything at all in order to influence your weight or shape?	1. 0 days<br>2. 1-2 days<br>3. 3-5 days<br>4. 6-7 days
99	Has thinking about food, eating or calories made it very difficult to concentrate on things you are interested in (such as working, following a conversation or reading)?	1. 0 days<br>2. 1-2 days<br>3. 3-5 days<br>4. 6-7 days
100	Has thinking about your weight or shape made it very difficult to concentrate on things you are interested in (such as working, following a conversation or reading)?	1. 0 days<br>2. 1-2 days<br>3. 3-5 days<br>4. 6-7 days
101	Have you had a definite fear that you might gain weight?	1. 0 days<br>2. 1-2 days<br>3. 3-5 days<br>4. 6-7 days
102	Have you had a strong desire to lose weight?	1. 0 days<br>2. 1-2 days<br>3. 3-5 days<br>4. 6-7 days

**103**	Have you tried to control your weight or shape by making yourself sick (vomit) or taking laxatives?	1. 0 days<br>2. 1-2 days<br>3. 3-5 days<br>4. 6-7 days
**104**	Have you exercised in a driven or compulsive way to control your weight, shape or body fat, or to burn off calories?	1. 0 days<br>2. 1-2 days<br>3. 3-5 days<br>4. 6-7 days
**105**	Have you felt like you lost control over your eating (at the time you were eating)?	1. 0 days<br>2. 1-2 days<br>3. 3-5 days<br>4. 6-7 days
**106**	**ONLY IF 1-2 DAYS, 3-5 DAYS OR 6-7 DAYS SELECTED FOR Q105**<br>On how many of these days (i.e. days on which you had a sense of having lost control over your eating) did you eat what other people would regard as an unusually large amount of food in one go?	1. 0 days<br>2. 1-2 days<br>3. 3-5 days<br>4. 6-7 days
**107**	Has your weight or shape influenced how you think about (judge) yourself as a person?	1. 0 days<br>2. 1-2 days<br>3. 3-5 days<br>4. 6-7 days
**108**	How dissatisfied have you been with your weight or shape?	1. 0 days<br>2. 1-2 days<br>3. 3-5 days<br>4. 6-7 days

Oral Health

In this part of the survey, you will be asked questions about your oral health. Remember, if you do not want to answer a question, you can skip it.

Item No.	Items	Response format
109	How often do you brush your teeth?	1. More than once a day<br>2. Once a day<br>3. At least once a week but not daily<br>4. Less than once a week<br>5. Never
110	Do you have a dentist that you see every six months or so?	1. No<br>2. Not sure<br>3. Yes

Puberty

In this part of the survey, you will be asked questions about your experience of going through puberty and some of the changes your body may go through. Remember, all of your responses are completely confidential, and if you do not want to answer a question, you can skip it.

Item No.	Items	Response format	
111	Puberty can invovle lots of changes to your body such as a growth spurt, new body hair, changes to your body, skin or voice. These changes happen for different people at different ages. Has your body started changing?	1. I have not started puberty<br>2. I have only just started puberty<br>3. I have definitely started puberty<br>4. I have completed puberty	
112	**ONLY IF FEMALE SELECTED FOR Q21**<br>How old were you when you had your first period? Please enter your age in years.	YES	NO

01274 274474
ageofwonder@bthft.nhs.uk
www.borninbradford.nhs.uk

BiB AGE OF WONDER

NHS Bradford Teaching Hospitals NHS Foundation Trust

SECTION 5 – HEALTH BEHAVIOURS

Smoking and Vaping

Remember, all of your responses are completely confidential. If you do not want to answer a question, you can skip it.

Item No.	Items	Response format	
113	Have you ever smoked cigarettes? (not including vape/e-cigarettes)	YES	NO
114	**ONLY IF YES SELECTED FOR Q113**<br>If yes, how often do you smoke cigarettes?	1. I have only ever tried smoking once or twice<br>2. I used to smoke but don't any more<br>3. I smoke one day a week or less<br>4. I smoke a few days a week<br>5. I smoke every day or nearly every day	
115	Have you ever vaped/used an e-cigarette?	YES	NO
116	**ONLY IF YES SELECTED FOR Q115**<br>Please read the following statements carefully and decide which ONE best describes you.	1. I have only ever tried vaping once or twice<br>2. I used to vape but don't any more<br>3. I vape one day a week or less<br>4. I vape a few days a week<br>5. I vape every day or nearly every day	

Alcohol

Remember, all of your responses are completely confidential. If you do not want to answer a question, you can skip it.

Item No.	Items	Response format	
117	Have you ever had an alcoholic drink that is more than a few sips? A drink is half a pint of lager, one alcopop, a small glass of wine, or a measure of spirits.	YES	NO
118	**ONLY IF YES SELECTED FOR Q115**<br>How old were you when you first had an alcoholic drink?	Answer age in years	
119	**ONLY IF YES SELECTED FOR Q115**<br>How many times have you had an alcoholic drink in the last 12 months? If you have had more than one alcoholic drink at a time, count this as one time.	1. Never<br>2. 1-2 times<br>3. A few times<br>4. Monthly<br>5. Weekly<br>6. More than once a week	

120	**ONLY IF YES SELECTED FOR Q115**<br>Have you ever had 5 or more alcoholic drinks at a time? A drink is a half pint of lager, beer or cide, one alcopop, a small glass of wine, or a measure of spirits.	YES	NO
121	**ONLY IF YES SELECTED FOR Q120**<br>How old were you when you first had 5 or more alcoholic drinks at a time?	Answer age in years	
122	**ONLY IF YES SELECTED FOR Q120**<br>How many times have you had 5 or more alcoholic drinks at a time n the last 12 months?	1. Never<br>2. 1-2 times<br>3. A few times<br>4. Monthly<br>5. Weekly<br>6. More than once a week	

Drugs

The next few questions are about drugs. This means drugs taken for fun (NOT including cigarettes, vapes, and alcohol, or medication prescribed to you). Remember, all of your answers are completely confidential and if you do not want to answer a question, you can skip it.

Item No.	Items	Response format	
123	Have you ever taken drugs?	YES	NO
124	**ONLY IF SELECTED YES TO Q123**<br>Have you ever taken any of the following? Cannabis (also called weed, marjuana, spliff, bud, zoot, whacky, baccy, hash or edibles)?	YES	NO
125	**ONLY IF SELECTED YES TO Q124**<br>In the past year how many times have you taken cannabis?	1. Once or twice<br>2. Three or four times<br>3. Five to ten times<br>4. More than ten times<br>5. Not taken in last year	
126	**ONLY IF SELECTED YES TO Q123**<br>Have you ever taken any of the following? Cocaine powder (also called coke, cowie or sniff)	YES	NO

BiB AGE OF WONDER

NHS Bradford Teaching Hospitals
NHS Foundation Trust

127	**ONLY IF SELECTED YES TO Q126**<br>In the past year how many times have you taken cocaine?	1. Once or twice<br>2. Three or four times<br>3. Five to ten times<br>4. More than ten times<br>5. Not taken in last year	
128	**ONLY IF SELECTED YES TO Q123**<br>Have you every taken any of the following? Ecstasy (also called 'E', MDMA or pills)	YES	NO
129	**ONLY IF SELECTED YES TO Q128**<br>In the past year how many times have you taken ecstasy?	1. Once or twice<br>2. Three or four times<br>3. Five to ten times<br>4. More than ten times<br>5. Not taken in last year	
130	**ONLY IF SELECTED YES TO Q123**<br>Have you every taken any of the following? Ketamine (also called ket)	YES	NO
131	**ONLY IF SELECTED YES TO Q130**<br>In the past year how many times have you taken ketamine?	1. Once or twice<br>2. Three or four times<br>3. Five to ten times<br>4. More than ten times<br>5. Not taken in last year	
132	**ONLY IF SELECTED YES TO Q123**<br>Have you every taken any of the following? Spice	YES	NO
133	**ONLY IF SELECTED YES TO Q132**<br>In the past year how many times have you taken Spice?	1. Once or twice<br>2. Three or four times<br>3. Five to ten times<br>4. More than ten times<br>5. Not taken in last year	
134	**ONLY IF SELECTED YES TO Q123**<br>Have you every taken any of the following? Nitrous Oxide (also called nos/noz, laughing gas, balloons, nitty)	YES	NO

135	**ONLY IF SELECTED YES TO Q134**<br>In the past year how many times have you taken Nitrous Oxide?	1. Once or twice<br>2. Three or four times<br>3. Five to ten times<br>4. More than ten times<br>5. Not taken in last year	
136	**ONLY IF SELECTED YES TO Q123**<br>Have you every taken any of the following? Prescription drugs not prescribed to you (for example codeine, tramadol, morphine, benzos)	YES	NO
137	**ONLY IF SELECTED YES TO Q136**<br>In the past year how many times have you taken prescription drugs for recreational use?	1. Once or twice<br>2. Three or four times<br>3. Five to ten times<br>4. More than ten times<br>5. Not taken in last year	
138	**ONLY IF SELECTED YES TO Q123**<br>Have you every taken any of the following? Any other drug not listed above.	YES	NO
139	**ONLY IF SELECTED YES TO Q138**<br>Any other drug (please specify):	Text box	
140	**ONLY IF SELECTED YES TO Q138**<br>In the past year how many times have you taken this other drug?	1. Once or twice<br>2. Three or four times<br>3. Five to ten times<br>4. More than ten times<br>5. Not taken in last year	

Gambling

The next few questions are about gambling activities you might have taken part in. Gambling includes gaming, betting and lotteries. Remember, everything you tell us is confidential no one at school will see your answers and if you do not want to answer a question, you can skip it.

Item No.	Items	Response format
141	Have you ever spend money on any of the following activities? Please tick all that apply.	1. Lotto (the main National Lottery draw) National Lottery Scratchcards which you bought in a shop (not free Scratchcards). Any other National Lottery games (e.g. EuroMillions, Thunderball, HotPicks, Set For Life). Other Lotteries (e.g. The Health Lottery, People's Postcode Lottery, or other smaller lotteries).<br>2. Fruit or slot machines (e.g. at an arcade, pub or social club).<br>3. Placing a private bet for money (e.g. with friends or family)<br>4. Playing cards for money (e.g. with friends or family).<br>5. Bingo at a bingo club.<br>6. Bingo at somewhere other than a bingo club (e.g. social club, holiday park, etc.)<br>7. Personally visiting a betting shop to play gaming machines.<br>8. Personally placing a bet at a betting shop (e.g. on football, dog racing or horse racing).<br>9. Personally visiting a casino to play casino games (by this we mean a proper casino with roulette tables) can win real money (e.g. poker, casinos, bingo, betting on sport or racing).<br>10. Gambling websites/apps where you can win real money (e.g. poker, casinos, bingo, betting on sport or racing).

142	**ONLY IF SELECTED NUMBER 1 TO Q141**<br><br>When did you last spend money on Lotto (the main National Lottery draw)<br>National Lottery Scratchcards which you bought in a shop (not free Scratchcards)<br>Any other National Lottery games (e.g. EuroMillions, Thunderball, HotPicks, Set For Life) Other Lotteries (e.g. The Health Lottery, People's Postcode Lottery, or other smaller lotteries)?	1. In the last 7 days<br>2. In the last month<br>3. In the last year<br>4. More than a year ago
143	**ONLY IF SELECTED NUMBER 2 TO Q141**<br><br>When did you last spend money on fruit or slot machines (e.g. at an arcade, pub or social club)?	1. In the last 7 days<br>2. In the last month<br>3. In the last year<br>4. More than a year ago
144	**ONLY IF SELECTED NUMBER 3 TO Q141**<br>When did you last spend money placing a private bet for money (e.g. with friends or family)?	1. In the last 7 days<br>2. In the last month<br>3. In the last year<br>4. More than a year ago
145	**ONLY IF SELECTED NUMBER 4 TO Q141**<br>When did you last spend money playing cards for money (e.g. with friends or family)?	1. In the last 7 days<br>2. In the last month<br>3. In the last year<br>4. More than a year ago
146	**ONLY IF SELECTED NUMBER 5 TO Q141**<br>When did you last spend money on bingo at a bingo club?	1. In the last 7 days<br>2. In the last month<br>3. In the last year<br>4. More than a year ago
147	**ONLY IF SELECTED NUMBER 6 TO Q141**<br>When did you last spend money on Bingo at somewhere other than a bingo club (e.g. social club, holiday park, etc.)	1. In the last 7 days<br>2. In the last month<br>3. In the last year<br>4. More than a year ago
148	**ONLY IF SELECTED NUMBER 7 TO Q141**<br>When did you last spend money personally visiting a betting shop to play gaming machines?	1. In the last 7 days<br>2. In the last month<br>3. In the last year<br>5. More than a year ago
149	**ONLY IF SELECTED NUMBER 8 TO Q141**<br>When did you last spend money personally placing a bet at a betting shop (e.g. on football, dog racing or horse racing)?	1. In the last 7 days<br>2. In the last month<br>3. In the last year<br>4. More than a year ago

150	**ONLY IF SELECTED NUMBER 9 TO Q141**<br>When did you last spend money personally visiting a casino to play casino games (by this we mean a proper casion with roulette tables)?	1. In the last 7 days<br>2. In the last month<br>3. In the last year<br>4. More than a year ago
151	**ONLY IF SELECTED NUMBER 10 TO Q141**<br>When did you last spend money on gambling websites/ apps where you can win real money (e.g. poker, casinos, bingo, betting on sport or racing)?	1. In the last 7 days<br>2. In the last month<br>3. In the last year<br>4. More than a year ago
152	As far as you know, has anyone in your immediate family (parent, siblings, other relatives you live with, or someone else who is responsible for looking after you) spent money on gambling?	1. Never – as far as you know<br>2. Yes – in the last 12 months<br>3. Yes – more than 12 months ago<br>4. Don't know<br>5. Prefer not to say

Online Games

When playing video games on a computer, console, streaming or mobile app (e.g. Fortnite, FIFA, Roblox, Candy Crush) it is sometimes possible to collect in-game items (e.g. skins, clothes, weapons, accessories, players).

Item No.	Items	Response format
153	In which, if any, of the following ways have you personally ever used in-game items or currecy? Tick all that apply	1. Paid money (or used virtual currency you have bought) to buy specific in-game items (e.g. skins, clothes, weapons, players).<br>2. Paid money (or used virtual currency you have bought) to open loot boxes/packs/chests to get other in-game items (e.g. skins, clothes, weapons, players).<br>3. Bet with in-game items on websites outside of the game you are playing.<br>4. None of these

Knives

In this part of the survey, you will be asked questions about knives. Remember, your response is confidential and you won't get into trouble for any of your answers. If you do not want to answer a question, you may skip it.

Item No.	Items	Response format	
154	In the last 12 months have you carried a knife or other weapon? For your own protection, because someone else asked you to or in case you get into a fight.	YES	NO

Police Contact

In this part of the survey, you will be asked questions about your experiences with the police. Remember, your response is confidential, and nobody will know how you have answered. If you do not want to answer a question, you can skip it.

Item No.	Items	Response format	
155	Have you ever been stopped and questioned by the police?	YES	NO
156	Have you ever been given a formal warning or caution by a police officer?	YES	NO

01274 274474
ageofwonder@bthft.nhs.uk
www.borninbradford.nhs.uk

Bradford Teaching Hospitals
NHS Foundation Trust

Section 6 – PHYSICAL HEALTH II

Physical Activity

VALIDATED MEASURE: Physical Activity Questionnaire for Adolescents (PAQA) (subset)

We are asking about your level of physical activity from the last 7 days (in the last week). This includes sport or dance that makes you sweat, makes your legs feel tired, or makes you breathe hard. For example, running, football, cycling etc.

Item No.	Items	Response format
157	In the last 7 days, on how many mornings did you actively travel to school? For example walking, cycling, scootering, and skateboarding.	1. None<br>2. 1 time last week<br>3. 2 or 3 times last week<br>4. 4 times last week<br>5. 5 times last week
158	In the last 7 days, during your physical edcuation (PE) classes, how often were you very active (playing hard, running, jumping, throwing?	1. I don't do PE<br>2. Hardly ever<br>3. Sometimes<br>4. Quite often<br>5. Always
159	In the last 7 days, what did you normally do at lunch (besides eating lunch)?	1. Sat down (talking, reading, doing school work)<br>2. Stood around or walked around<br>3. Ran or played a little bit<br>4. Ran and played hard most of the time
160	In the last 7 days, on how many afternoons did you actively travel (for example, walking, cycling, scootering and skateboarding) from school?	1. None<br>2. 1 time last week<br>3. 2 or 3 times last week<br>4. 4 times last week<br>5. 5 times last week
161	In the last 7 days, on how many days right after school, did you do sports, dance, or play games in which you were very active?	1. None<br>2. 1 time last week<br>3. 2 or 3 times last week<br>4. 4 times last week<br>5. 5 times last week
162	In the last 7 days, oh how many evenings did you do sports, dance, or play games in which you were very active?	1. None<br>2. 1 time last week<br>3. 2 or 3 times last week<br>4. 4 or 5 times last week<br>5. 6 or 7 times last week

163	On the last weekend, how many times did you do sports, dance, or play games in which you were very active?	1. None<br>2. 1 time<br>3. 2-3 times<br>4. 4-5 times<br>5. 6 or more times	
164	Were you sick last week, or did anything prevent you from doing your normal physical activies?	YES	NO
165	**ONLY IF YES SELECTED FOR Q164**<br>What prevented you?	Text box	

Sedentary Activity

VALIDATED MEASURE: Youth Activity Profile (sedentary scale only)

These questions are about sedentary activity out of school. Sedentary activity means activity sitting or lying down, including things like watching TV, playing video games, and using a mobile phone.

Item No.	Items	Response format
	**Please select how long you usually spend doing the following activities:**	
166	Watching TV outside of school time. This includes time spent watching TV programmes, movies or sports but NOT time spent playing video games.	1. Did not do<br>2. Less than 1 hour per day<br>3. 1 to 2 hours per day<br>4. 2 to 3 hours per day<br>5. 3 hours per day or more
167	Playing video games outside of school time. This includes games on things like Nintendo DS, wii, Xbox, PlayStation, iPod Touch, iPad, or games on your PC and phone.	1. Did not do<br>2. Less than 1 hour per day<br>3. 1 to 2 hours per day<br>4. 2 to 3 hours per day<br>5. 3 hours per day or more
168	Using computers or tablets outside of school time. This doesn't include homework time and playing online video games or computer games, but does not include time on things like Instagram and Facebook, as well as time spent on the internet, and messaging like Whatsapp.	1. Did not do<br>2. Less than 1 hour per day<br>3. 1 to 2 hours per day<br>4. 2 to 3 hours per day<br>5. 3 hours per day or more
169	Using a mobile phone after school. This includes time spent talking, texting, or using things like social media but does not include playing games.	1. Did not do<br>2. Less than 1 hour per day<br>3. 1 to 2 hours per day<br>4. 2 to 3 hours per day<br>5. 3 hours per day or more

170	Which of the following best describes your typical sedentary habits at home? Try to think about a typical week and not just last week.	1. I spend almost none of my free time sitting<br>2. I spend little time sitting during my free time<br>3. I spend about half of my free time sitting<br>4. I spend a lot of time sitting during my free time<br>5. I spend almost all of my free time sitting

Sleep

We would like to know about the time you go to sleep and wake up.

Item No.	Items	Response format
	**Please think about the last 7 days when answering these questions:**	
171	On school nights, what time do you typically fall asleep at?	Drop down: Time
172	On school days, what time do you typically wake up for the day at?	Drop down: Time
173	On non-school nights, what time do you typically fall asleep at?	Drop down: Time
174	On non-school days, what time do you typically wake up for the day at?	Drop down: Time
175	Do you sleep well at night?	1. No<br>2. Sometimes<br>3. Yes, always
176	How likely are you to feel sleepy during the day?	1. Rarely<br>2. Sometimes<br>3. Often
