# Supplementary material for: Ethnic and income-related inequalities in mental health among adolescents: a cross-sectional study in Bradford, UK
Source: BMJ Public Health. 2026 Sep 15;4(3):e004528. doi: 10.1136/bmjph-2025-004528 (PMC13583858; doi:10.1136/bmjph-2025-004528)
Supplement: Supplementary file 3 [file bmjph-4-3-s003.pdf]

Bradford Teaching Hospitals
NHS Foundation Trust

BiB AGE OF WONDER

MODULE TWO (232)
2023-24

MENTAL HEALTH
ENVIRONMENT
SCHOOL
IDENTITY & DISCRIMINATION
DIGITAL & SOCIAL MEDIA

01274 274474
ageofwonder@bthft.nhs.uk
www.borninbradford.nhs.uk

Doc46_mod232_infographic_v1_4.8.23

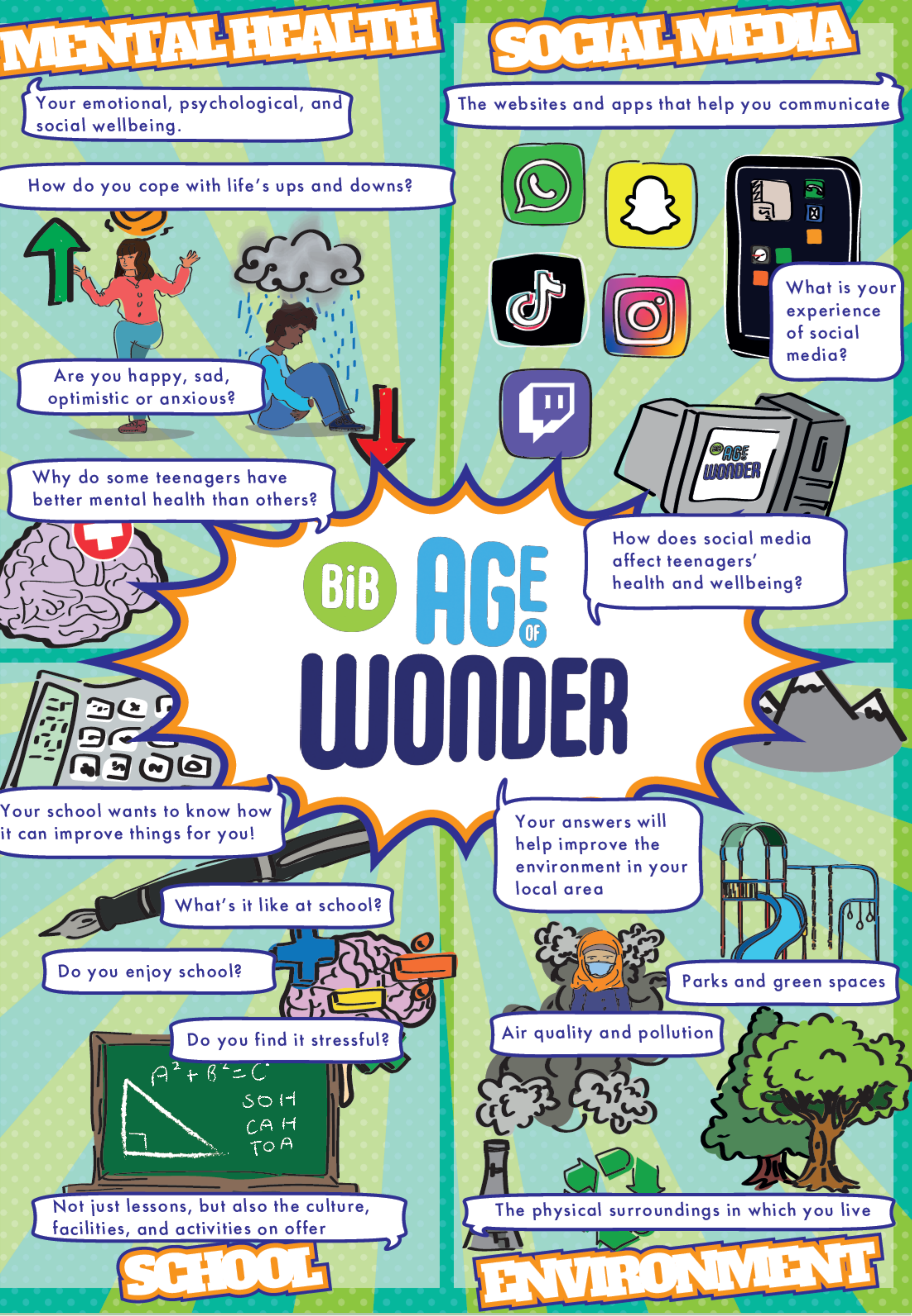

Age of Wonder

Bradford Teaching Hospitals NHS Foundation Trust

CONTENTS

SECTION 1 – MENTAL HEALTH AND WELLBEING

Mental ill health

Relationships

Trust

Self-harm

Resilience

Unusual Experiences

SECTION 2 – ENVIRONMENT

Green space

Pollution

Climate change

SECTION 3 – SCHOOL

Behaviour

School pressure

Friends

01274 274474

ageofwonder@bthft.nhs.uk

www.borninbradford.nhs.uk

CONTENTS

SECTION 4 – IDENTITY AND DISCRIMINATION

Bullying

Discrimination

SECTION 5 – DIGITAL AND SOCIAL MEDIA

Social media

Internet

01274 274474

ageofwonder@bthft.nhs.uk

www.borninbradford.nhs.uk

BiB AGE OF WONDER

NHS Bradford Teaching Hospitals NHS Foundation Trust

Item No.	Items	Response format		
1	Participant Study ID	Text		
2	Year Group	8	9	10

SECTION 1 – MENTAL HEALTH AND WELLBEING

Mental Ill Health YEAR 8 AND 10 ONLY

VALIDATED MEASURE: Revised Children's Anxiety and Depression Scale – 25 item version (RCADS-25)

In this part of the survey, you will be asked questions about your mental ill health. Some of these questions may be difficult to answer. Remember, all of your responses are completely confidential and if you do not want to answer a question, you can skip it. If anything upsets you, please talk to your teacher or access the information provided at the end of the survey. Please select how often each of these things happen to you. There are no right or wrong answers.

Item No.	Items	Response format
	**Please select how often these things happen to you. There are no right or wrong answers.**	
3	I feel sad or empty	1. Never<br>2. Sometimes<br>3. Often<br>4. Always
4	I worry when I think that I have done poorly at something	1. Never<br>2. Sometimes<br>3. Often<br>4. Always
5	I would feel afraid of being on my own at home.	1. Never<br>2. Sometimes<br>3. Often<br>4. Always

BiB AGE OF WONDER

NHS
Bradford Teaching Hospitals
NHS Foundation Trust

6	Nothing is much fun anymore	1. Never<br>2. Sometimes<br>3. Often<br>4. Always
7	I worry that something awful will happen to someone in my family	1. Never<br>2. Sometimes<br>3. Often<br>4. Always
8	I am afraid of being in crowded places (like shopping centres, the movies, buses, busy playgrounds)	1. Never<br>2. Sometimes<br>3. Often<br>4. Always
9	I worry what other people think of me	1. Never<br>2. Sometimes<br>3. Often<br>4. Always
10	I have trouble sleeping	1. Never<br>2. Sometimes<br>3. Often<br>4. Always
11	I feel scared if I have to sleep on my own	1. Never<br>2. Sometimes<br>3. Often<br>4. Always
12	I have problems with my appetite	1. Never<br>2. Sometimes<br>3. Often<br>4. Always
13	I suddenly become dizzy or faint when there is no reason for this	1. Never<br>2. Sometimes<br>3. Often<br>4. Always
14	I have to do some things over and over again (like washing my hands, cleaning or putting things in a certain order)	1. Never<br>2. Sometimes<br>3. Often<br>4. Always

15	I have no energy for things	1. Never<br>2. Sometimes<br>3. Often<br>4. Always
16	I suddenly start to tremble or shake when there is no reason for this	1. Never<br>2. Sometimes<br>3. Often<br>4. Always
17	I cannot think clearly	1. Never<br>2. Sometimes<br>3. Often<br>4. Always
18	I feel worthless	1. Never<br>2. Sometimes<br>3. Often<br>4. Always
19	I have to think special thoughts (like numbers or words) to stop bad things happening	1. Never<br>2. Sometimes<br>3. Often<br>4. Always
20	I think about death	1. Never<br>2. Sometimes<br>3. Often<br>4. Always
21	I feel like I don’t want to move	1. Never<br>2. Sometimes<br>3. Often<br>4. Always
22	I worry that I will suddenly get a scared feeling when there is nothing to be afraid of	1. Never<br>2. Sometimes<br>3. Often<br>4. Always
23	I am tired a lot	1. Never<br>2. Sometimes<br>3. Often<br>4. Always

ageofwonder@bthft.nhs.uk
www.borninbradford.nhs.uk

24	I feel afraid that I will make a fool of myself in front of people	1. Never<br>2. Sometimes<br>3. Often<br>4. Always
25	I have to do some things in just the right way to stop bad things from happening	1. Never<br>2. Sometimes<br>3. Often<br>4. Always
26	I feel restless	1. Never<br>2. Sometimes<br>3. Often<br>4. Always
27	I worry that something bad will happen to me	1. Never<br>2. Sometimes<br>3. Often<br>4. Always

Strengths and Difficulties YEAR 9 ONLY

VALIDATED MEASURE: Strengths and Difficulties Questionnaire (SDQ)

In this part of the survey, you will be asked questions about your emotional strengths and difficulties. Some of these questions may be difficult to answer. For each item, please mark the box for Not True, Somewhat True or Certainly True. It would help us if you answered all items as best you can even if you are not absolutely certain, or the item seems daft! Please give your answers on the basis of how things have been for you over the last six months.

Item No.	Items	Response format
	**Please give your answers on the basis of how things have been for you over the last six months.**	
28	I try to be nice to other people. I care about their feelings	1. Not true<br>2. Somewhat true<br>3. Certainly true
29	I am restless, I cannot stay still for long	1. Not true<br>2. Somewhat true<br>3. Certainly true

30	I get a lot of headaches, stomaches or sickness	1. Not true<br>2. Somewhat true<br>3. Certainly true
31	I usually share with others (food, games, pens etc)	1. Not true<br>2. Somewhat true<br>3. Certainly true
32	I get very angry and often loose my temper	1. Not true<br>2. Somewhat true<br>3. Certainly true
33	I am usually on my own. I generally play alone or keep to myself	1. Not true<br>2. Somewhat true<br>3. Certainly true
34	I usually do as I am told	1. Not true<br>2. Somewhat true<br>3. Certainly true
35	I worry a lot	1. Not true<br>2. Somewhat true<br>3. Certainly true
36	I am helpful if someone is hurt, upset or feeling ill	1. Not true<br>2. Somewhat true<br>3. Certainly true
37	I am constantly fidgeting or squirming	1. Not true<br>2. Somewhat true<br>3. Certainly true
38	I have one good friend or more	1. Not true<br>2. Somewhat true<br>3. Certainly true
39	I fight a lot. I can make other people do what I want	1. Not true<br>2. Somewhat true<br>3. Certainly true
40	I am often unhappy, down-hearted or tearful	1. Not true<br>2. Somewhat true<br>3. Certainly true

41	Other people my age generally like me	1. Not true<br>2. Somewhat true<br>3. Certainly true
42	I am easily distracted I find it difficult to concentrate	1. Not true<br>2. Somewhat true<br>3. Certainly true
43	I am nervous in new situations. I easily loose confidence	1. Not true<br>2. Somewhat true<br>3. Certainly true
44	I am kind to younger children	1. Not true<br>2. Somewhat true<br>3. Certainly true
45	I am often accused of lying or cheating	1. Not true<br>2. Somewhat true<br>3. Certainly true
46	Other children or young people pick on me or bully me	1. Not true<br>2. Somewhat true<br>3. Certainly true
47	I often volunteer to help other (parents, teachers, children)	1. Not true<br>2. Somewhat true<br>3. Certainly true
48	I think before I do things	1. Not true<br>2. Somewhat true<br>3. Certainly true
49	I take things that are not mine from home, school or elsewhere	1. Not true<br>2. Somewhat true<br>3. Certainly true
50	I get on better with adults than with people my own age	1. Not true<br>2. Somewhat true<br>3. Certainly true
51	I have many fears, I am easily scared	1. Not true<br>2. Somewhat true<br>3. Certainly true

52	I finish the work I am doing. My attention is good.	1. Not true<br>2. Somewhat true<br>3. Certainly true

Wellbeing

VALIDATED MEASURE: Short Warwick-Edinburgh Mental Wellbeing Scale (SWEMWBS)

In this part of the survey, you will be asked questions about your wellbeing. Remember, if you do not want to answer a question, you can skip it.

Item No.	Items	Response format
	**Below are statements about feelings and thoughts. Please tick the box that best describes your experience of each over the past 2 weeks**	
53	I've been feeling optimistic about the future	1. None of the time<br>2. Rarely<br>3. Some of the time<br>4. Often<br>5. All the time
54	I've been feeling useful	1. None of the time<br>2. Rarely<br>3. Some of the time<br>4. Often<br>5. All the time
55	I've been feeling relaxed	1. None of the time<br>2. Rarely<br>3. Some of the time<br>4. Often<br>5. All the time
56	I've been dealing with problems well	1. None of the time<br>2. Rarely<br>3. Some of the time<br>4. Often<br>5. All the time

57	I've been thinking clearly	1. None of the time<br>2. Rarely<br>3. Some of the time<br>4. Often<br>5. All the time
58	I've been feeling close to other people	1. None of the time<br>2. Rarely<br>3. Some of the time<br>4. Often<br>5. All the time
59	I've been able to make up my own mind about things	1. None of the time<br>2. Rarely<br>3. Some of the time<br>4. Often<br>5. All the time
60	When I find something really hard, I can work out what to do	1. None of the time<br>2. Rarely<br>3. Some of the time<br>4. Often<br>5. All the time

Relationships

This part of the survey will ask you questions about your relationships. This includes questions about your social circle and your peers. Remember, if you do not want to answer a question, you can skip it.

Item No.	Items	Response format
	**How often do you feel...**	
61	...that you lack friendships?	1. Hardly ever<br>2. Some of the time<br>3. Often
62	...left out?	1. Hardly ever<br>2. Some of the time<br>3. Often

63	...isolated from others?	1. Hardly ever<br>2. Some of the time<br>3. Often
64	...alone?	1. Hardly ever<br>2. Some of the time<br>3. Often

Trust

Item No.	Items	Response format
65	Generally speaking, would you say that most people can be trusted, or you can't be too careful in dealing with other people?	1. Most people can be trusted<br>2. Can't be too careful

Help Seeking

VALIDATED MEASURE: General Help Seeking Questionnaire (GHSQ)

Item No.	Items	Response format
	**If you had a personal or emotional problems, how likely is it you would seek help from the following?**	
66	Girlfriend/boyfriend or partner	1. 1 (Extremely unlikely)<br>2. 2<br>3. 3 (Unlikely)<br>4. 4<br>5. 5 (Likely)<br>6. 6<br>7. 7 (Extremely likely)<br>8. I don't have a girlfriend, boyfriend or partner
67	Friend (not related to you)	1. 1 (Extremely unlikely)<br>2. 2<br>3. 3 (Unlikely)<br>4. 4<br>5. 5 (Likely)<br>6. 6<br>7. 7 (Extremely likely)

68	Parent/carer	1. 1 (Extremely unlikely)<br>2. 2<br>3. 3 (Unlikely)<br>4. 4<br>5. 5 (Likely)<br>6. 6<br>7. 7 (Extremely likely)
69	Mental health professional (e.g. psychologist, social worker, counsellor)	1. 1 (Extremely unlikely)<br>2. 2<br>3. 3 (Unlikely)<br>4. 4<br>5. 5 (Likely)<br>6. 6<br>7. 7 (Extremely likely)
70	Phone helpine (e.g. lifeline/samaritans/NSPCC)	1. 1 (Extremely unlikely)<br>2. 2<br>3. 3 (Unlikely)<br>4. 4<br>5. 5 (Likely)<br>6. 6<br>7. 7 (Extremely likely)
71	Doctor/GP	1. 1 (Extremely unlikely)<br>2. 2<br>3. 3 (Unlikely)<br>4. 4<br>5. 5 (Likely)<br>6. 6<br>7. 7 (Extremely likely)
72	Religious Leader	1. 1 (Extremely unlikely)<br>2. 2<br>3. 3 (Unlikely)<br>4. 4<br>5. 5 (Likely)<br>6. 6<br>7. 7 (Extremely likely)

73	Teacher or other school staff member	1. 1 (Extremely unlikely)<br>2. 2<br>3. 3 (Unlikely)<br>4. 4<br>5. 5 (Likely)<br>6. 6<br>7. 7 (Extremely likely)	
74	I would not seek help from anyone	1. 1 (Extremely unlikely)<br>2. 2<br>3. 3 (Unlikely)<br>4. 4<br>5. 5 (Likely)<br>6. 6<br>7. 7 (Extremely likely)	
75	Would you seek help from another not listed above?	YES	NO
76	**ONLY IF ANSWERED YES TO Q75**<br>Please list in the space provided:	Text box	

TW: Self-Harm

Please remember all of your responses are completely confidential and will not be shared with your teachers, friends, or parents. If you do not want to answer a question you can skip it.

Item No.	Items	Response format	
77	In the past year, have you hurt yourself on purpose in any way?	YES	NO

Resilience **YEAR 8 ONLY**

VALIDATED MEASURE: Brief Resilience Scale (BRS)

Item No.	Items	Response format
	**Please indicate how much you agree or disagree with the following statements:**	
78	I tend to bounce back quickly after hard times	1. Strongly Disagree<br>2. Disagree<br>3. Neutral<br>4. Agree<br>5. Strongly Agree
79	I have a hard time making it through stressful events	1. Strongly Disagree<br>2. Disagree<br>3. Neutral<br>4. Agree<br>5. Strongly Agree
80	It is hard for me to snap back when something bad happens	1. Strongly Disagree<br>2. Disagree<br>3. Neutral<br>4. Agree<br>5. Strongly Agree
81	I usually come through difficult times with little troublew	1. Strongly Disagree<br>2. Disagree<br>3. Neutral<br>4. Agree<br>5. Strongly Agree
82	I tend to take a long time to get over set-backs in my life	1. Strongly Disagree<br>2. Disagree<br>3. Neutral<br>4. Agree<br>5. Strongly Agree

Unusual Experiences YEAR 10 ONLY

Here we ask some questions about some unusual experiences you may have had. It can be quite normal to hear things that other people don't. Sometimes people may have strange beliefs or feel a bit out of touch with reality, this can feel upsetting sometimes, but it is quite common. You can find some services in the BiB resources booklet that might be able to help if any of these questions are upsetting.

Item No.	Items	Response format
83	Have you ever heard voices that other people couldn't hear?	1. Yes, defintely<br>2. Yes, maybe<br>3. No, never
84	**ONLY IF ANSWERED 1 OR 2 TO Q83**<br>At its worst, how upsetting did you find this?	1. Not at all upset<br>2. A bit upsetting<br>3. Quite upsetting<br>4. Very upswetting
85	**ONLY IF ANSWERED 1 OR 2 TO Q83**<br>How often have you heard voices that other people couldn't hear in the last year?	1. Once or twice<br>2. Less than once a month<br>3. More than once a month<br>4. Nearly every day<br>5. Not at all
86	Have you ever seeen something or someone that other people could not see?	1. Yes, defintely<br>2. Yes, maybe<br>3. No, never
87	**ONLY IF ANSWERED 1 OR 2 TO Q86**<br>At its worst, how upsetting did you find this?	1. Not at all upset<br>2. A bit upsetting<br>3. Quite upsetting<br>4. Very upswetting
88	**ONLY IF ANSWERED 1 OR 2 TO Q86**<br>How often have you seen something or someone that other people couldn't in the past year?	1. Once or twice<br>2. Less than once a month<br>3. More than once a month<br>4. Nearly every day<br>5. Not at all
89	Have you ever thought you were being followed or spied on?	1. Yes, defintely<br>2. Yes, maybe<br>3. No, never
90	**ONLY IF ANSWERED 1 OR 2 TO Q89**<br>At its worst, how upsetting did you find this?	1. Not at all upset<br>2. A bit upsetting<br>3. Quite upsetting<br>4. Very upswetting
91	**ONLY IF ANSWERED 1 OR 2 TO Q89**<br>How often have you thought you were being followed or spied on in the past year?	1. Once or twice<br>2. Less than once a month<br>3. More than once a month<br>4. Nearly every day<br>5. Not at all

92	**ONLY IF ANSWERED 1 OR 2 TO Q89**<br>Have you ever believed that people were following you or spying on you as part of a plot to harm you in some way, and which your family or friends did not believe existed?	1. Yes, defintely<br>2. Yes, maybe<br>3. No, never
93	Some people believe that other people can read their thoughts. Have other people ever read your thoughts?	1. Yes, defintely<br>2. Yes, maybe<br>3. No, never
94	**ONLY IF ANSWERED 1 OR 2 TO Q93**<br>At its worst, how upsetting did you find this?	1. Not at all upset<br>2. A bit upsetting<br>3. Quite upsetting<br>4. Very upswetting
95	**ONLY IF ANSWERED 1 OR 2 TO Q93**<br>How often have you believed that other people can read your thoughts in the past year?	1. Once or twice<br>2. Less than once a month<br>3. More than once a month<br>4. Nearly every day<br>5. Not at all
96	Do you think people have sometimes used special powers to read your thoughts?	1. Yes, definitely<br>2. Yes, maybe<br>3. No, never
97	Have you ever believed that you were being sent special messages through the television or the radio, or that a programme had been arranged just for you alone?	1. Yes, defintely<br>2. Yes, maybe<br>3. No, never
98	**ONLY IF ANSWERED 1 OR 2 TO Q97**<br>At its worst, how upsetting did you find this?	1. Not at all upset<br>2. A bit upsetting<br>3. Quite upsetting<br>4. Very upswetting
99	**ONLY IF ANSWERED 1 OR 2 TO Q97**<br>How often have you been sent special messages in the past year?	1. Once or twice<br>2. Less than once a month<br>3. More than once a month<br>4. Nearly every day<br>5. Not at all
100	Have you ever felt that you were under the control of some special power?	1. Yes, definitely<br>2. Yes, maybe<br>3. No, never

101	**ONLY IF ANSWERED 1 OR 2 TO Q100**<br>At its worst, how upsetting did you find this?	1. Not at all upsetting<br>2. A bit upsetting<br>3. Quite upsetting<br>4. Very upsetting
102	**ONLY IF ANSWERED 1 OR 2 TO Q100**<br>How often have you thought you were under the control of a special power in the past year?	1. Once or twice<br>2. Less than once a month<br>3. More than once a month<br>4. Nearly everyday<br>5. Not at all
103	**ONLY IF ANSWERED 1 OR 2 TO Q100**<br>Who do you think was controlling you (at any time in the past?)	1. God<br>2. A computer/other machine<br>3. Someone/something else
104	**ONLY IF ANSWERED 1 OR 2 TO Q100**<br>Did it control what you were doing or thinking, such that you had no will of your own?	1. Yes, definitely<br>2. Yes, maybe<br>3. No, never
105	Have you ever felt that you are somebody realy very special, or that you have special powers like reading people's mind, or that you have been chosen to perform great and special tasks? (This does not mean that you are just clever or you come from an important family)	1. Yes, definitely<br>2. Yes, maybe<br>3. No, never
106	**ONLY IF ANSWERED 1 OR 2 TO Q105**<br>How often have you thought you are somebody really special, or that you have special powers in the past year?	1. Once or twice<br>2. Less than once a month<br>3. More than once a month<br>4. Nearly everyday<br>5. Not at all

01274 274474
ageofwonder@bthft.nhs.uk
www.borninbradford.nhs.uk

SECTION 2: ENVIRONMENT

Green Space

In this part of the survey, you will be asked questions about the green spaces in your area and your usage of them.

Item No.	Items	Response format
107	How often do you visit parks and green spaces? During the winter months (Septemer - March)	1. 5 times a week or more<br>2. 2-4 times a week<br>3. Once a week<br>4. 1-3 times a month<br>5. Less than once a month
108	How often do you visit parks and green spaces? During the spring and summer months (April-August)	1. 5 times a week or more<br>2. 2-4 times a week<br>3. Once a week<br>4. 1-3 times a month<br>5. Less than once a month

Pollution

In this part of the survey, you will be asked questions about air pollution and air quality.

Item No.	Items	Response format
109	What do you think about the air quality in Bradford genreally? Air quality is the term we use to describe how polluted the air we breathe is.	1. Very poor<br>2. Poor<br>3. Fair<br>4. Good<br>5. Excellent<br>6. Don't know

Climate change

In this part of the survey, you will be asked questions about climate change and your feelings about how it affects you

Item No.	Items	Response format
110	How positive do you currenlty feel when you think about the future of the environment?	1. Very positive<br>2. Fairly positive<br>3. Neither positive nor negative<br>4. Fairly negative<br>5. Very negative

SECTION 3 – SCHOOL

In this part of the survey you will be asked questions about your school. Remember, your answers are completely confidential so please be as honest as you can. If you do not want to answer a question, you can skip it.

Item No.	Items	Response format
111	I enjoy school	1. All the time<br>2. Most of the time<br>3. Some of the time<br>4. Almost never<br>5. Never
112	There is an adult at school I can talk to if something is worrying me	1. Strongly agree<br>2. Agree<br>3. Neither agree nor disagree<br>4. Disagree<br>5. Strongly disagree

Behaviour

In this part of the survey, you will be asked questions about how people behave at school. Remember, nobody will know how you've responded, and if you do not want to answer a question, you can skip it.

Item No.	Items	Response format
113	Is bullying a problem at your school?	1. It doesn't happen<br>2. It happens and teachers are really good at resovling it<br>3. It happens and teachers are good at resolving it<br>4. It happens and teachers are not good at resolving it<br>5. It happens and teachers do nothing about it

114	I feel safe when I'm at school	1. All the time<br>2. Most of the time<br>3. Some of the time<br>4. Almost never<br>5. Never

School pressure

Item No.	Items	Response format
	**How much do you agree or disagree with the following statements...**	
115	Competition with other people for grades is intense	1. Strongly agree<br>2. Agree<br>3. Neither agree nor disagree<br>4. Disagree<br>5. Strongly disagree
116	If I don't do well in school, my family will be dissaponted.	1. Strongly agree<br>2. Agree<br>3. Neither agree nor disagree<br>4. Disagree<br>5. Strongly disagree
117	My teachers put too much pressure on me to do well in school.	1. Strongly agree<br>2. Agree<br>3. Neither agree nor disagree<br>4. Disagree<br>5. Strongly disagree
118	I'm worried about progressing in the future	1. Strongly agree<br>2. Agree<br>3. Neither agree nor disagree<br>4. Disagree<br>5. Strongly disagree
119	If I don't do well in school, I'll be a failure	1. Strongly agree<br>2. Agree<br>3. Neither agree nor disagree<br>4. Disagree<br>5. Strongly disagree

120	I often feel stressed because of the pressure to do well in school	1. Strongly agree<br>2. Agree<br>3. Neither agree nor disagree<br>4. Disagree<br>5. Strongly disagree
121	I worry about doing well in tests, exams and assessments	1. Strongly agree<br>2. Agree<br>3. Neither agree nor disagree<br>4. Disagree<br>5. Strongly disagree

Friends

Item No.	Items	Response format	
122	How many in-person friends do you have? By in-person we mean friends you've met in real life	1. None<br>2. Not many<br>3. Some<br>4. Lots	
123	How many online friends do you have? By online we mean friends you only know online	1. None<br>2. Not many<br>3. Some<br>4. Lots	
124	Do you have any close friends? By close friends we mean other young people you feel at ease with or who you can talk to about things that are private	YES	NO

SECTION 4: IDENTITY AND DISCRIMINATION

Attitudes and Values

In this part of the survey, you will be asked questions about your attitudes and values. Remember, if you do not want to answer a question, you can skip it.

Item No.	Items	Response format
	**How many people your age would....**	
125	Start a fight with somone?	1. None of them<br>2. Some of them<br>3. Most of them<br>4. All of them
126	Write things or spray paint a building, fence or train?	1. None of them<br>2. Some of them<br>3. Most of them<br>4. All of them
127	Take something from a shop without paying?	1. None of them<br>2. Some of them<br>3. Most of them<br>4. All of them
128	Copy or downlaod music, games, or films without paying for them, when they should have done?	1. None of them<br>2. Some of them<br>3. Most of them<br>4. All of them

Bullying

In this part of the survey, you will be asked questions about bullying. We say a person is being bullied when another person or a group of people, repeatedly say or do unwanted nasty and unpleasant things to them. It also is bullying when a person is teased in a way they do not like or when they are left out of things on purpose. The person that bullies has more power than the person being bullied and wants to cause harm to them. Your answers will help to identify where bullying is occurring and inform the design of supports and services for those being bullied. Remember, your answers are completely confidential and will not be shared with your school, your friends, or family members. If you do not want to answer a question, you can skip it.

Item No.	Items	Response format
129	How often have you taken part in bullying another person(s) at school in the past couple months?	1. I have not bullied another person(s) in the past couple of months.<br>2. It has happened once or twice<br>3. 2 or 3 times a month<br>4. About once a week<br>5. Several times a week (3-5 times)<br>6. Everyday
130	How often have you been bullied at school in the past couple of months?	1. I have not been bullied at school in the past couple of months<br>2. It has happened once or twice<br>3. 2 or 3 times a month<br>4. About once a week<br>5. Several times a week (3-5 times)<br>6. Everyday
131	In the past couple of months, how often have you taken part in online bullying? (Using a phone, device or computer to bully someone through messages, gaming or a social media platform)	1. I have not bullied another person(s) online in the past couple of months.<br>2. It has happened once or twice<br>3. 2 or 3 times a month<br>4. About once a week<br>5. Several times a week (3-5 times)<br>6. Everyday
132	In the past couple of months, how often have you been bullied online? (Been bullied by someone using a phone, device or computer to bully you through messages, gaming or a social media platform)	1. I have not been bullied online in the past couple of months.<br>2. It has happened once or twice<br>3. 2 or 3 times a month<br>4. About once a week<br>5. Several times a week (3-5 times)<br>6. Everyday

Discrimination

VALIDATED MEASURE: Adolescent Discrimination Distress Index (ADDI) (adapted)

In this part of the survey, you will be asked questions about discrimination. We would like to know about any experiences you have had where you have experienced discrimination because of your ethnicity, your sex/gender identity, disability, religion, class, neurodiversity or other reasons. We are interested in if and how people treat you differently because of these reasons. Remember, all of your responses are confidential, so please answer the questions you feel comfortable with.

Item No.	Items	Response format	
**Have you experienced any of the following:**			
133	You were discouraged from joining a club or group.	YES	NO
134	Others your age did not include you in their activities	YES	NO
135	People expected less of you than they expected of others your age.	YES	NO
136	People assumed your English was poor.	YES	NO
137	You were hassled by police.	YES	NO
138	You were hassled by staff in a shop.	YES	NO
139	You were called insulting names.	YES	NO
140	You received poor service in a shop, restaurant, or similar place.	YES	NO
141	People acted as though you were not intelligent.	YES	NO
142	People acted as if they were afraid of you.	YES	NO
143	You were threatened	YES	NO

01274 274474
ageofwonder@bthft.nhs.uk
www.borninbradford.nhs.uk

144	**ANSWER ONLY IF YES TO Q133-143**<br>If you had this experience, was it because of your:	Tick all that apply:<br>1. Ethnicity<br>2. Sex/ gender identity<br>3. Disability<br>4. Religion<br>5. Class<br>6. Neurodiversity)e.g. conditions such as 7.Autism, Asperger's, Dyslexia)<br>8. Sexuality<br>9. Other
145	**ANSWER ONLY IF TICKED OTHER TO Q144**<br>Please describe	Text box

SECTION 4: DIGITAL AND SOCIAL MEDIA

Social Media

In this part of the survey, you will be asked questions about social media and your positive and negative experiences with it. Your answers will help researchers and local organisations understand teenagers' social media habits and support positive experiences online. Remember, your responses are completely confidential and will not be shared with any teachers, friends, or family members. If you do not want to answer a question, you can skip it.

Item No.	Items	Response format
146	Which social media platforms do you use? Tick all that apply.	1. Facebook<br>2. Instagram<br>3. X (formerly Twitter)<br>4. TikTok<br>5. Snapchat<br>6. Other
147	**ANSWER ONLY IF TICKED OTHER TO Q146**<br>Please describe	Text box
148	On a normal week day (Monday-Friday) during term time, roughly how many hours do you spend using social media?	Drop down: hours
149	What has been your positive experiences of using social media? Tick all that apply.	1. Connecting with friends<br>2. Viewing enjoyable content<br>3. Learning new things<br>4. Accessing support<br>5. Engaging positively with current issues<br>6. Other

01274 274474
ageofwonder@bthft.nhs.uk
www.borninbradford.nhs.uk

150	**ONLY IF TICKED OTHER TO Q149**<br>Please describe	Text box
151	What have been your negative experiences of using social media? Tick all that apply.	1. People saying or writing unpleasant things about you (with words, pictures or video)<br>2. People bullying you<br>3. Pictures, videos or games with violence you found upsetting<br>4. Sexually explicit images, videos or games<br>5. Posting or sending something online you wish you hadn't (text, images, videos)<br>6. I haven't experienced any of these<br>7. Other
152	**ONLY IF TICKED OTHER TO Q151**<br>Please describe	Text box
153	Has your experience of using social media been mostly positive or negative?	1. All positive<br>2. Mostly positive<br>3. An even mix of positive and negative<br>4. Mostly negative<br>5. All negative

Internet

In this section we talk about the internet. This means content you can only access online such as, social media, websites, and online games.

Item No.	Items	Response format	
154	Do you have access to the internet at home?	YES	NO
155	**ONLY IF YES TO Q154**<br>How good is your internet at home?	1. Very bad<br>2. Bad<br>3. Okay<br>4. Good<br>5. Very good	

01274 274474
ageofwonder@bthft.nhs.uk
www.borninbradford.nhs.uk
